# Paracrine Signals from the Tumor Microenvironment Modulate Stem Cell Collagen Interaction and Remodeling, Indicating Early Functional Changes

**DOI:** 10.3390/cells15191775

**Published:** 2026-09-29

**Authors:** Lora Topalova, Tanya Stoyanova, Nadia García-Parra, José Luis Gómez Ribelles, Svetla Todinova, George Altankov

**Affiliations:** 1 Institute of Biophysics and Biomedical Engineering, Bulgarian Academy of Sciences, 1113 Sofia, Bulgaria; topalovaloram@gmail.com (L.T.); tanya.zh.stoyanova@gmail.com (T.S.); todinova@abv.bg (S.T.); 2 Center of Competence in Personalized Medicine, 3D and Telemedicine, Robotic Assisted and Minimally Invasive Surgery—“Leonardo da Vinci”, 5800 Pleven, Bulgaria; 3 Centre for Biomaterials and Tissue Engineering, Polytechnic University of Valencia, 46022 Valencia, Spain; nadiagarciaparra1999@gmail.com (N.G.-P.); jlgomez@ter.upv.es (J.L.G.R.); 4 Biomedical Research Center Networking in Bioengineering, Biomaterials and Nanomedicine (CIBER-BBN), 28029 Madrid, Spain; 5 Associate Project BG-RRP-2.004-0003, Medical University Pleven, 5800 Pleven, Bulgaria; 6 Research Institute, Medical University Pleven, 5800 Pleven, Bulgaria

**Keywords:** tumor microenvironment, mesenchymal stem cells (MSCs), cancer cell secretome, MSC collagen interaction and remodeling, multiple myeloma and B-cell leukemia cell lines

## Abstract

The tumor microenvironment (TME) is shaped by dynamic interactions between malignant cells and stromal components, with mesenchymal stem cells (MSCs) contributing to extracellular matrix (ECM) remodeling. Here, we examined how secretomes from two hematological cancer cell lines, RPMI-8226 (multiple myeloma) and HG-3 (B-cell leukemia), influence early functional responses of human adipose-derived MSCs (AD-MSCs) and their interaction with and reorganization of collagen. Cancer-conditioned media accelerated MSC proliferation (~2-fold faster doubling time) and increased SA-β-galactosidase activity (~3–4-fold above control). Upon adhesion to collagen, treated MSCs displayed distinct morphologies and reduced cell spreading (~2100 μm^2^ vs. ~2500 μm^2^ in controls), accompanied by altered collagen organization. To quantify collagen remodeling, we introduced the collagen removal index (CRI), expressing reorganized collagen area relative to cell spreading area. CRI revealed higher remodeling efficiency in HG-3-treated MSCs than in RPMI-8226-treated and control cells, indicating enhanced collagen-interacting capacity. Secretome exposure also modulated cytokine secretion, with increased bFGF under both conditions and IL-6 upregulation specifically following HG-3 exposure. After secretome withdrawal, most functional alterations, including senescence-associated changes, motility, and elevated bFGF, persisted. Notably, IL-6 upregulation and increased CRI emerged in RPMI-8226-exposed MSCs after withdrawal, suggesting a delayed response. Together, these findings reveal early, partially persistent functional shifts in MSC behavior and collagen remodeling that may represent an early stromal response with potential relevance to tumor progression.

## 1. Introduction

Mesenchymal stromal/stem cells (MSCs) are key regulators of tissue homeostasis and repair, owing to their multipotent regenerative potential, immunomodulatory properties and capacity to remodel the extracellular matrix (ECM) [1]. In recent years, increasing attention has been directed toward the remarkable plasticity of MSCs within pathological environments, particularly in the context of cancer.

The concept that tumors can recruit and reprogram MSCs into a pro-tumorigenic stromal population emerged in the early 2000s, when MSCs were first shown to home to sites of inflammation and neoplasia [2,3]. In this work, we refer to this cell population as cancer-associated MSCs (CA-MSCs), even though the terms tumor-associated MSCs (TA-MSCs) and transformed MSCs are also widely used in the literature [4,5].

Subsequent studies demonstrated that once within the tumor microenvironment, MSCs undergo profound transcriptional, epigenetic, and functional reprogramming, acquiring immunosuppressive, angiogenic, and metastasis-supporting properties [6,7,8,9]. These findings established CA-MSCs as key architects of the tumor niche, capable of shaping malignant progression through reciprocal interactions with cancer cells. In this context, CA-MSCs help organize the tumor niche and modulate cancer progression [10,11], whereas cancer-associated fibroblasts (CAFs), which have also been extensively studied, provide a similar and potentially complementary stromal influence. The relationship between MSCs and CAFs remains unresolved, largely because no universally accepted CAF-specific markers have been established [4,12,13,14].

Tumors actively reshape their microenvironment through a complex mixture of soluble factors, extracellular vesicles, cytokines, and matrix-modifying enzymes, collectively recognized as the cancer cell secretome [15]. These paracrine signals can profoundly alter the phenotype and the function of resident or recruited stromal cells, including MSCs, driving their conversion into CA-MSCs or CAFs [16,17,18]. Nonetheless, CA-MSCs have emerged as critical contributors to tumor progression. They promote cancer cell proliferation, angiogenesis, immune evasion, and metastatic dissemination through secretion of pro-tumorigenic cytokines, remodeling of the ECM, and modulation of cancer cell mechanics [19]. Senescence is a particularly intriguing outcome of tumor-induced MSC reprogramming. While senescent MSCs exhibit reduced proliferative capacity, they often display a senescence-associated secretory phenotype (SASP) that enhances inflammation, ECM degradation, and tumor progression [20,21,22]. Notably, SASP arises from persistent cellular stress that activates senescence-driven secretory programs and denotes a senescence-associated shift in which stromal cells release elevated pro-inflammatory cytokines (like IL-6, IL-1, TNF-α), chemokines (like IL-8, CCL2, CXCL1, CXCL2), growth factors (like HGF, VEGF, bFGF), and matrix remodeling enzymes as well as reactive oxygen species (ROS) [23,24]. Paradoxically, several studies report that tumor-derived factors can simultaneously stimulate MSC proliferation and induce stress-related senescence markers, suggesting a dynamic transition from a proliferative burst to stress-induced premature senescence (SIPS) [25]. This dual behavior reflects the complex interplay between mitogenic and stress-inducing signals within the tumor microenvironment. This highlights the central role of the cancer cell secretome in shaping stromal cell behavior.

Hematological malignancies such as multiple myeloma and B-cell leukemias exhibit a pronounced dependence on their microenvironment, where stromal cells promote tumor survival, therapeutic resistance, and immune evasion [26,27,28]. Myeloma cells, for instance, release interleukin (IL) 6, transforming growth factor beta (TGF-β), vascular endothelial growth factor (VEGF) and exosomal microribonucleic acids (miRNAs) that reprogram bone-marrow-derived MSCs (BM-MSCs) toward a senescent, pro-inflammatory and ECM-remodeling phenotype [29,30]. Likewise, leukemic B-cells secrete soluble mediators capable of altering MSC proliferation, migration, differentiation, and matrix-degrading activity [31,32,33]. Given this strong niche dependency within the pathological bone-marrow environment, an important question is whether malignant hematologic cells exert comparable effects on MSCs from non-marrow tissues, such as adipose-derived MSCs (AD-MSCs). The presence of extramedullary myeloma and leukemic infiltration into adipose-rich tissues further suggests that AD-MSCs may represent alternative stromal targets relevant to disease dissemination. Although extensive work has established the contribution of MSC-derived factors to tumor progression, increasing evidence indicates that cancer-cell-derived secretomes can also reprogram MSCs toward tumor-associated and cancer-associated fibroblast-like phenotypes, particularly in solid-tumor models [34,35]. In multiple myeloma, several studies have demonstrated that myeloma cells, conditioned media, or myeloma-derived extracellular vesicles can alter the phenotype, gene expression, secretory activity, and functional properties of bone-marrow-derived MSCs [36,37,38]. Adipose-derived MSCs have been investigated in the context of multiple myeloma, including their interaction with myeloma cells and their altered properties in patients with MM [39,40], but we found no previous study specifically characterizing the effects of a multiple-myeloma-derived secretome on healthy human AD-MSC phenotype or ECM/collagen-remodeling behavior. As for chronic lymphocytic leukemia, Paggetti et al. demonstrated that CLL-derived extracellular vesicles are taken up by MSCs and induce a cancer-associated fibroblast-like phenotype, providing evidence for EV-mediated reprogramming of stromal cells [32]; however, whether CLL-derived secretome components similarly affect adipose-derived MSCs has not been specifically investigated. More broadly, previous studies of tumor-mediated stromal activation have predominantly characterized molecular and biochemical endpoints, including transcriptional changes in CAF and ECM-associated genes, expression or activity of matrix-remodeling enzymes, and proteomic alterations [34,41]. Although tumor-secretome signaling and ECM remodeling are recognized hallmarks of tumor–stroma communication, the mechanical remodeling of collagen by MSCs after indirect exposure to cancer-cell secretomes has not been previously quantified. This is particularly relevant given that tumor cells can reshape adjacent collagen matrices through both enzymatic degradation and mechanical reorganization, processes that facilitate their spread within the tissue. To our knowledge, this is the first study to delineate how hematologic cancer-cell secretomes drive mechanical collagen remodeling by MSCs and to compare the distinct effects of multiple myeloma versus chronic lymphocytic leukemia secretomes on adipose-derived MSCs. This contribution is particularly important because many clinical-grade MSC products originate from adipose or umbilical-cord tissue. Therefore, defining how malignant secretomes reprogram these MSC populations has direct implications for the safety, stability, and therapeutic design of MSC-based interventions.

The aim of this study is to determine how cancer cell secretomes derived from two hematological cancer cell lines: RPMI-8226 (multiple myeloma) and HG-3 (B-cell leukemia), modulate key functional properties of human AD-MSCs. To evaluate the impact of these paracrine signals on AD-MSCs, we applied a functional, systems-level approach, focusing on phenotypic and functional alterations while also examining correlations with elevated levels of bFGF and IL-6, present in the conditioned media. This strategy enabled us to distinguish immediate from persistent stromal responses following secretome exposure. We evaluated changes in cell adhesion and spreading, proliferation, senescence-associated features, and ECM remodeling using FITC-collagen as a model substrate. We further examined whether these tumor-induced phenotypic alterations persist after removal of the secretome, providing insight into the stability and durability of MSC reprogramming. Collectively, these analyses illuminate early events in CA-MSC formation and reveal new aspects of stromal involvement in cancer progression.

## 2. Materials and Methods

### 2.1. Cells and Culturing

#### 2.1.1. Cancer Cell Lines

The human multiple myeloma cell line RPMI-8226 (American Type Culture Collection, ATCC, Manassas, VA, USA) and the human chronic lymphocytic leukemia cell line HG-3 (purchased by Leibniz Institute DSMZ-German Collection of Microorganisms and Cell Cultures, Braunschweig, Germany) were cultured in RPMI-1640 medium supplemented with 10% fetal bovine serum (FBS), 1% penicillin-streptomycin, and 2 mM L-glutamine (all from Sigma Aldrich, St. Louis, MO, USA) in standard T25 or T75 tissue culture (TC) flasks (Corning, Glendale, AZ, USA) at a seeding density of 5 × 10^5^ cells/mL and cultured under standard conditions (37 °C, 5% CO_2_).

#### 2.1.2. Human Adipose Tissue-Derived Mesenchymal Stem Cells

Human AD-MSCs used for the experiments were obtained and characterized by Tissue Bank BulGen (Sofia, Bulgaria) with informed consent from donors prior to liposuction. MSCs used in this study were derived from three independent human donors, and the reported results are representative of observations consistently obtained across all three donors. Cells were cultured in DMEM/F12 (Cat. № D8437) medium supplemented with 10% FBS (Cat. № F7524, Lot:0001665006) and 1% antibiotic-antimycotic solution (all from Sigma-Aldrich, St. Louis, MO, USA) using small T25 TC flasks (Greiner Bioone, Meckenheim, Germany) in a humidified thermostat at 37 °C, 5% CO_2_. Medium (typically 5 mL) was replaced every 2 to 3 days until cells reached ~90% confluency and then passaged using 0.05% trypsin/0.6 mM EDTA (Sigma-Aldrich, USA). Cells used for experiments were between passages 4 and 6.

### 2.2. Accumulation of Cancer Cell Secretome

Cancer cell secretomes were obtained from the human multiple myeloma cell line RPMI-8226 and the human chronic lymphocytic leukemia cell line HG-3 under serum-free conditions. Serum-free collection was selected to minimize contamination of the cancer-cell secretome with serum-derived proteins and growth factors that could otherwise interfere with the subsequent analysis and biological assays. The cells were washed with phosphate-buffered saline (PBS) (Sigma-Aldrich, USA) by centrifugation at 400× *g* and seeded at a density of 5 × 10^5^ cells in 5 mL serum-free RPMI 1640 medium with 1% penicillin-streptomycin and 2 mM L-glutamine (all from Sigma-Aldrich, USA) before being incubated for 72 h in serum-free conditions to allow accumulation of soluble factors released by the cancer cells. Serum-free medium was used for technical isolation of tumor-derived paracrine factors.

Following incubation, the conditioned media were collected and cleared by centrifugation at 400× *g* for 10 min to remove cell debris. The resulting supernatant was subsequently concentrated 10 times using Amicon Ultra centrifugal filters (Amicon, Schorndorf, Germany) with a 3 kDa molecular weight cutoff, according to the manufacturer’s instructions. Fresh cell media was subjected to the same treatment as the conditioned media, to be used later as a control. After concentration, the protein content of the cancer-cell secretome was quantified by Bradford assay and was approximately 0.5 mg/mL. The concentrated secretome was subsequently diluted 1:10 in the culture medium used for AD-MSC treatment, resulting in a final protein concentration of approximately 0.05 mg/mL. Thus, the 0.05 mg/mL concentration represents the estimated protein concentration corresponding to the original conditioned medium of cancer cells. Concentrated secretomes and empty controls were aliquoted and stored at −80 °C until use.

### 2.3. Treatment with Cancer Cell Secretome

AD-MSCs were seeded in 6-well TC plates (Sensoplate, Greiner Bioone, Meckenheim, Germany) at a density of 3.3 × 10^4^ cells/well corresponding to 3.4 × 10^3^ cells/cm^2^ in 2 mL DMEM/F12 with 1% antibiotic–antimycotic solution, 10% FBS (all from Sigma-Aldrich, as stated above) and RPMI-8226/HG-3 secretome was diluted 1:10 in culture medium, resulting in a final protein concentration of approximately 0.05 mg/mL. For a control, cells were treated with 10× diluted control media concentrate (as detailed in Section 4.2). The cells were cultured in standard conditions (37 °C, 5% CO_2_) for 3 days to assess the direct “Exposure” effects. This interval minimizes variability from media changes and provides sufficient time for secretome-driven functional and morphological responses to develop [42,43].

For the evaluation of the stability of these effects (e.g., the “Withdrawal” outcome), the cells were washed 2 times with PBS and further replaced with secretome-free media for an additional 3 days. The initial seeding density for these conditions was 1.7 × 10^4^ cells/well, corresponding to 1.8 × 10^3^ cells/cm^2^. The lower seeding density for the “Withdrawal” group was used to compensate for the additional three days of cell growth prior to treatment [42,43].

### 2.4. Live-Dead Analysis

AD-MSCs were seeded in 6-well tissue-culture plates (Sensoplate, Greiner Bio-One, Meckenheim, Germany) at a density of 3.3 × 10^4^ cells/well (3.4 × 10^3^ cells/cm^2^) for the “Exposure” samples and 1.7 × 10^4^ cells/well (1.8 × 10^3^ cells/cm^2^) for the “Withdrawal” samples. Cells were then exposed to secretome derived from RPMI-8226 or HG-3 cells at a final protein concentration of 0.05 mg/mL or to control medium supplemented with 10% FBS and 1% antibiotic-antimycotic solution (all reagents from Sigma-Aldrich, USA). “Withdrawal” samples were handled identically for the first three days but then switched to secretome-free medium for three more days.

Cell viability was assessed at 24 h and 72 h using the Cellstain Double Staining Kit (Sigma-Aldrich, Cat. No. 04511) following the manufacturer’s instructions. A freshly prepared staining solution containing Calcein-AM (labeling viable cells in green) and propidium iodide (PI) (labeling nuclei of non-viable cells in red) was added to each well at a ratio of 10 μL per 1 mL of medium. Samples were incubated for 15 min at 37 °C in the dark prior to imaging. Fluorescent images were acquired using a fluorescence microscope (Thunder Imager Live Cell, Leica Microsystems, Heerbrugg, Switzerland) with appropriate filter sets to count the percentage of live versus dead cells.

### 2.5. Overall Cell Morphology

To investigate the overall cell morphology after treatment (under “Exposure” and “Withdrawal” conditions), AD-MSCs were seeded in 24-well glass-bottomed plates (Greiner Bio-One, Frickenhausen, Germany) pre-coated with collagen (see Section 2.10) at a density of 1 × 10^4^ cells/well (5.3 × 10^3^ cells/cm^2^) and cultured for 2 h in serum-free medium to isolate collagen-specific attachment and early focal adhesion formation, after which 10% FBS was added for an additional 3 h to support adhesion stabilization and cell spreading.

Then the samples were fixed with 4% paraformaldehyde (PFA) and permeabilized with 0.5% Triton X-100 for 5 min before being washed and treated with blocking solution (10% FBS dissolved in PBS) for 15 min. The actin cytoskeleton was visualized using Atto 633 phalloidin (Sigma-Aldrich, USA), and the nuclei were counterstained with Hoechst 33258 (Sigma-Aldrich, 1:2000 dilution). Fluorescent images were acquired using a fluorescence microscope (Thunder Imager, as above) with a 20× objective. To determine the cell spreading area quantitatively, the CellProfiler software version 4.2.8 [44] was used.

### 2.6. Cell Proliferation Assay

AD-MSCs were treated as described in Section 4.3. To evaluate cell proliferation, samples were analyzed after 24, 48 and 72 h of culture. At each time point, cells were fixed with 4% PFA and stained with Hoechst 33258 (1:2000 dilution) to visualize nuclei. Fluorescence images of the entire wells were acquired at 10× magnification using a Thunder fluorescent microscope as above. Quantification was performed with CellProfiler software, enabling objective counting of nuclei across nine fields per condition from three independent experiments. Cell doubling time was calculated using the following formula:

Doubling Time = [T × (ln2)]/[ln(Ne/Nb)]

where Nb is the initial cell density, Ne is the final cell density, and T is the time passed between the two measurements.

### 2.7. Cellular Senescence-Associated SA β-gal Activity

AD-MSCs were treated with tumor secretome as described in Section 4.3. Following the incubation period, cells were assessed using the Senescence Cells Histochemical Staining Kit (CS0030) (Sigma-Aldrich, USA). The staining procedure was performed according to the manufacturer’s instructions. After completion of the staining protocol, cells were rinsed with PBS, and the percentage of senescence-associated β-galactosidase-positive cells was evaluated by light microscopy (Olympus CX43, Tokyo, Japan).

### 2.8. Cell Cycle Analysis

Cell cycle distribution was assessed following the protocol described by Vassilis Roukos [45]. Fluorescent images were acquired using a fluorescence microscope equipped with a 10× objective. The image set was then processed using the analysis pipeline provided by the Roukos laboratory, which enabled quantification of DNA content for each nucleus. First, an illumination-correction function was generated and applied to each image to compensate for uneven field illumination and background intensity gradients, ensuring that measured nuclear fluorescence reflected true signal rather than positional artifacts. After correction, nuclei were identified as primary objects based on their fluorescence borders, and the total integrated intensity of each nucleus was measured. These intensity values were exported as .csv files and reformatted to match the standard flow-cytometry data structure for downstream interpretation as DNA-content estimates. For each experimental condition, a minimum of 600 nuclei were evaluated.

Cell cycle analysis was performed using the flow cytometry analysis tool available at Floreada.io (https://floreada.io/, accessed on 24 April 2025). The nuclear intensity data were uploaded to the platform, where the standard Michael H. Fox cell cycle analysis algorithm [46] was applied. Based on the resulting DNA content profiles, nuclei were categorized into three cell cycle phases (G1/G0, S, and G2/M).

### 2.9. FITC-Labeling of Collagen

Collagen type I was isolated from rat tail tendon (RTC) by acetic-acid extraction followed by NaCl-induced salting-out, as described elsewhere [47]. The RTC concentration in the resulting solutions was determined by measuring optical absorbance at 220–230 nm. Labeling of RTC collagen with fluorescein isothiocyanate (FITC) was performed using a modified version of the protocol by Doyle [48]. Briefly, RTC (2 mg/mL) was dissolved in 0.05 M borate buffer (pH 8), after which 20 µg of FITC from a 1 mg/mL dimethyl sulfoxide (DMSO) stock solution was added. The mixture was incubated for 90 min at room temperature in the dark. The reaction was stopped with 0.05 M Tris buffer (pH 7.4), followed by extensive dialysis against 0.05 M acetic acid to remove unbound FITC. The molar FITC-to-protein ratio (F/P) was calculated from the UV-Vis spectrum of FITC-RTC using the adapted formula:

F/P = F/C = (Amax × D)/ε_0_ × CM

where Amax is the absorbance of the FITC-RTC solutions measured at 494 nm; D is a dilution factor; ε_0_ is the molar extinction coefficient of FITC, equal to 70,000 M^−1^ cm^−1^; CM is the molar collagen concentration.

### 2.10. Preparation of Collagen-Coated Surfaces

FITC-collagen was dissolved in 0.5 M acetic acid to a final concentration of 100 µg/mL and centrifuged at 13,000× *g* for 15 min, and the supernatant was collected. Then 24-well glass-bottom plates (Greiner Bio-One, Frickenhausen, Germany) were coated with 300 μL of the collagen solution and incubated at 37 °C for 1 h, followed by three rinses with phosphate-buffered saline (PBS).

### 2.11. Collagen Remodeling Assessment

#### 2.11.1. Remodeling upon Direct Contact with Cancer Cells

HG-3 and RPMI-8226 were seeded onto the fluorescent collagen substrate prepared as above (Section 2.10) in the 24-well glass-bottom plates at a density of 4 × 10^4^ cells/well (2.1 × 10^4^ cells/cm^2^) and allowed to attach for 2 h in serum-free medium. After the incubation, the media were collected and centrifuged at 400× *g* for 5 min. This removed cell debris for later analysis. The attached cells were fixed with 4% paraformaldehyde and stained with Hoechst 33258 (1:2000 dilution) and Atto 633 phalloidin to visualize nuclei and actin cytoskeleton, respectively. Imaging was performed using the inverted fluorescence microscope Leica DM 2900 (Leica Microsystems, Heerbrugg, Switzerland) at 20× magnification. Fluorescence of the supernatants was evaluated at 490/515 nm using the fluorometer Jasco FP-8050, and the acquired signal is presented as relative fluorescence units (RFU).

#### 2.11.2. Cancer Cell Secretome-Induced Effect on AD-MSC Collagen Remodeling

After completion of either the “Exposure” or “Withdrawal” protocols, AD MSCs were detached using 0.05% trypsin/0.6 mM EDTA (Sigma-Aldrich, USA) for approximately 5 min under standard culture conditions (37 °C, 5% CO_2_). Cells were then collected and seeded onto the fluorescent collagen substrate prepared as described in Section 2.8 and Section 2.9, at a density of 1 × 10^4^ cells/well (5.3 × 10^3^ cells/cm^2^) in 24-well glass-bottom plates, and allowed to attach for 2 h in serum-free medium.

Subsequently, 10% FBS was added, and the cells were further cultured for up to 5 h. Following incubation, cells were fixed with 4% paraformaldehyde and stained with Hoechst 33258 (1:2000 dilution) and Atto 633 phalloidin (1:100 dilution) to simultaneously visualize nuclei and actin cytoskeleton, respectively. Imaging was performed using the inverted fluorescence microscope Leica DM 2900 at 20× magnification. Image analysis was carried out using CellProfiler software version 4.2.8 [44] to quantify the dark regions corresponding to remodeled collagen within selected regions of interest (ROIs) surrounding the cells.

#### 2.11.3. Collagen Removal Index (CRI) Calculation

The collagen removal index (CRI) was used to quantify the extent of mechanical collagen rearrangement induced by individual cells. CRI was calculated from fluorescence images of collagen-coated substrates before and after cell interaction. First, the area of collagen cleared or displaced by each cell was segmented and measured as the absolute remodeled collagen area. The corresponding cell spreading area was determined for the same image. To account for differences in cell spreading, the remodeling activity was normalized to the corresponding cell area and expressed as the collagen removal index (CRI):

$$CRI=\frac{Remodeled\;collagen\;area}{Cell\;spreading\;area}$$

Absolute remodeled collagen area and cell spreading area were also analyzed separately.

#### 2.11.4. Endogenous Collagen Production

After the 5 h of incubation on RTC substratum, AD-MSCs were fixed with 4% paraformaldehyde and permeabilized using 0.5% Triton X-100 for 5 min. Cells were then washed and incubated in a blocking solution (PBS supplemented with 10% FBS; Sigma-Aldrich, USA) for 15 min. Subsequently, samples were incubated at 37 °C with rabbit anti-human collagen I antibody (AB745, Sigma-Aldrich, USA), followed by incubation with Alexa Fluor 555-conjugated donkey anti-rabbit IgG (406412, BioLegend, San Diego, CA, USA) as the secondary antibody. All antibodies were used at a 1:100 dilutions in PBS containing 10% FBS. The actin cytoskeleton was stained with Alexa Fluo 488 phalloidin (Invitrogen, Waltham, MA, USA), and nuclei were counterstained with Hoechst 33258 (1:2000). Fluorescent images were acquired using a Thunder Imager Live Cell microscope (Leica) with a 20× objective. Quantitative analysis of intracellular collagen I abundance and cell morphology was performed using CellProfiler software [44].

### 2.12. Artificial Wound-Healing (Scratch) Assay

AD-MSCs were seeded in 12-well plates (Sensoplate, Greiner Bio-One, Meckenheim, Germany) and cultured in 1 mL DMEM/F12 supplemented with 10% FBS and 1% antibiotic–antimycotic solution. For the “Exposure” protocol, the cancer cell secretome was added at a final protein concentration of 0.05 mg/mL, and the cells were incubated for 72 h under standard culture conditions. After incubation, a linear scratch was made across the cell monolayer using a sterile 200 µL pipette tip. The cells were then washed with DMEM/F12 media once and maintained in DMEM/F12, containing 10% FBS and 1% antibiotic-antimycotic solution. Time-lapse imaging was performed for 24 h using the live-cell chamber of the inverted fluorescence microscope (Thunder Imager Live Cell, Leica Microsystems, Switzerland), acquiring images at 10× magnification.

For the “Withdrawal” protocol, after 72 h of secretome exposure, the secretome-containing medium was removed, and the cells were washed twice with PBS before being supplied with fresh, secretome-free medium and cultured for an additional 3 days. The scratch assay was then performed as described above.

Wound-closure dynamics were quantified using ImageJ software version 1.54p (Wayne Rasband, National Institute of Mental Health, NIH, Bethesda, MD, USA) with the high-throughput scratch-assay analysis plugin [49]. The percentage of wound closure was calculated as:

%Closure = [(A_0_ − A_t_)/A_0_] × 100

where A_0_ is the wound area at 0 h and A_t_ is the area at time t.

### 2.13. Collection of AD-MSC Secretomes

AD-MSC secretomes were collected following the previously described “Exposure” and “Withdrawal” treatment protocols. At the end of each treatment period, the cells were thoroughly washed with phosphate-buffered saline (PBS; Sigma-Aldrich, USA) by centrifugation at 400× *g* to minimize carryover of treatment-derived factors. Subsequently, fresh serum-free DMEM supplemented with 1% penicillin-streptomycin and 2 mM L-glutamine (all from Sigma-Aldrich, USA) was added to the cells to initiate the secretome accumulation period. Cells were maintained for 48 h under standard culture conditions (5% CO_2_, 37 °C). Then the conditioned medium was collected and centrifuged at 400× *g* for 10 min to remove cellular debris. The resulting supernatants were subsequently concentrated 10-fold using Amicon Ultra centrifugal filters with a 3-kDa molecular weight cutoff (Amicon, Schorndorf, Germany), according to the manufacturer’s instructions. The concentrated secretome samples were aliquoted and stored at −80 °C until further analysis.

### 2.14. ELISA Quantification of IL-6 and FGF-Basic

IL-6 and basic FGF levels in conditioned media were quantified using the Human IL-6 ELISA Kit (Cat. No. 430507) and Human FGF-basic ELISA Kit (Cat. No. 434309). Secretomes from RPMI-8226 and HG-3 cancer cells, as well as from AD-MSCs treated with cancer-cell secretomes, were collected, cleared by centrifugation, and stored at −80 °C until analysis after a 10× concentration as described above.

All assays were performed according to the manufacturers’ protocols. Shortly, the standards and samples were added to pre-coated wells in duplicate, followed by incubation with detection antibodies and TMB substrate. Absorbance was measured at 450 nm with a reference wavelength of 570 nm. Cytokine concentrations were calculated using standard curves and expressed as pg/mL before concentration or as fold change relative to the corresponding untreated control condition.

### 2.15. Statistical Analysis

Statistical analyses were mostly performed using GraphPad Prism 8 (GraphPad Software, Boston, MA, USA). Data are presented as column bar graphs, line graphs and scatter plots with individual points representing independent replicates and are shown either as mean ± standard deviation (SD) or median and interquartile range (IQR). Normality was assessed using the Shapiro–Wilk test, which was followed by an evaluation of variance homogeneity with Bartlett’s test. Mauchly’s test was used to evaluate compliance with the sphericity assumption. The statistical tests applied, the corresponding significance levels, and the number of replicates are reported in the respective figures’ legends. For single-cell morphometric analyses, statistical analyses were performed in R (version 4.6.1). Cell-spreading area data were analyzed using linear mixed-effects model, with experimental condition included as a fixed effect and individual cell measurements as a random effect. Model assumptions were assessed using Q-Q plot of the model residual (Figure S1). Estimated marginal means (EMMs) were calculated and pairwise comparisons were performed using Tukey adjustment for multiple comparisons. Data are presented as violin plots, EMM and 95% confidence interval (CI).

## 3. Results

### 3.1. Cancer Cell Secretome-Induced Changes in Mesenchymal Stem Cells

**Figure 1:**
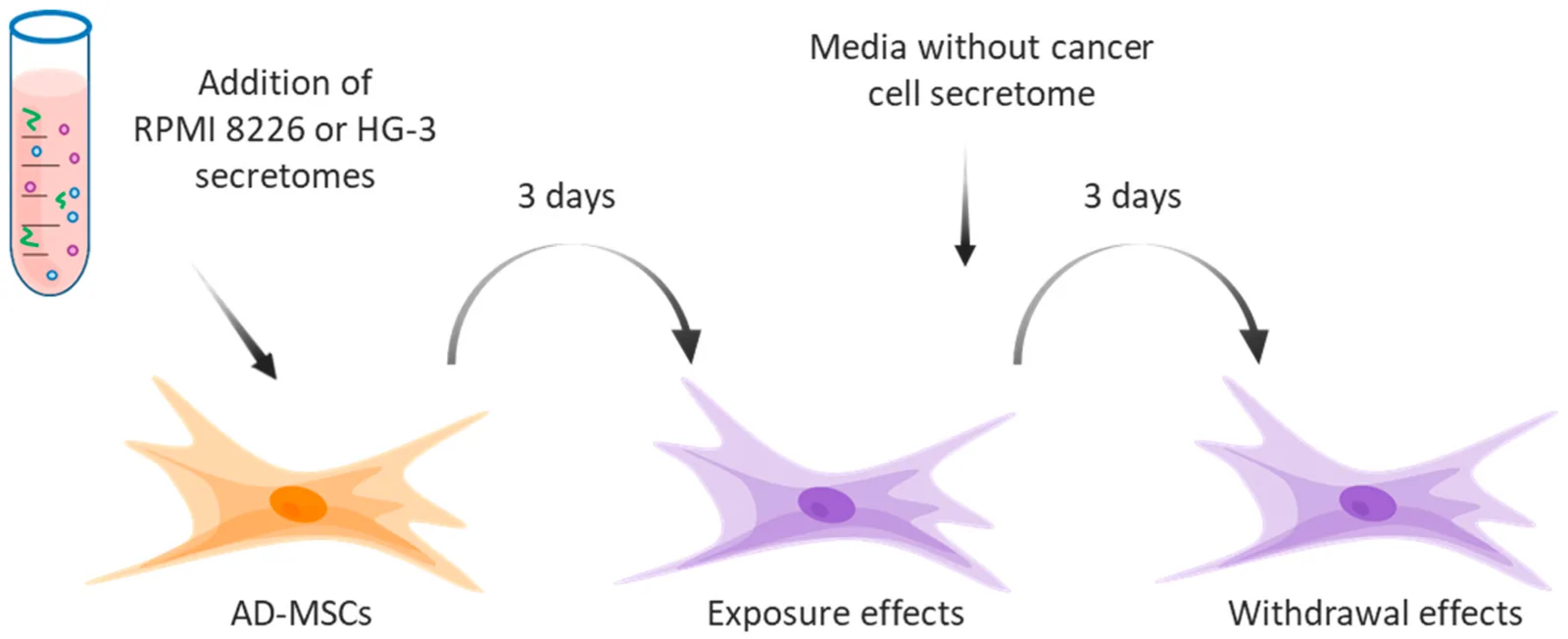
Schematic representation of the AD-MSCs cancer cell-secretome treatment setup. The concentrated RPMI-8226 or HG-3 secretomes were added to the cultured AD-MSCs and incubated for 3 days to evaluate the “Exposure” effects. For the “Withdrawal” effects, the AD-MSCs were further incubated for another 3 days in secretome-free medium.

Building on our main strategy we first examined how cancer-cell–derived secretomes alter AD-MSC functionality. Our analysis focused on early paracrine-driven shifts in MSC behavior upon their interaction with collagen, including alterations in adhesion, overall morphology, and substrate-collagen remodeling capacity. We also assessed key functional outcomes such as proliferation dynamics, migration, and senescence-associated responses. In addition, we show that secretome exposure is able to affect the levels of the stromal-modulating cytokines IL-6 and bFGF in MSC-conditioned media, further supporting the emergence of an activated state. Together, these alterations align with the initial stages of MSC activation described in the literature. Within this framework, the present study offers a quantitative assessment of early MSC functional change that precedes full CAF specification. To distinguish immediate from persistent effects, the experimental design incorporated two conditions: (i) MSCs cultured directly with cancer cell secretomes for 72 h (termed “Exposure”), allowing assessment of the direct paracrine influence of the secretome; and (ii) MSCs subsequently cultured for an additional 72 h in secretome-free medium (termed “Withdrawal”), enabling evaluation of lasting secretome-induced alterations (Figure 1). The data are presented in the corresponding graphs, which illustrate the magnitude and significance of the effects induced by both cancer cell secretomes.

#### 3.1.1. Morphology of AD-MSCs upon Indirect Contact with Cancer Cells on Collagen Substrate

**Figure 2:**
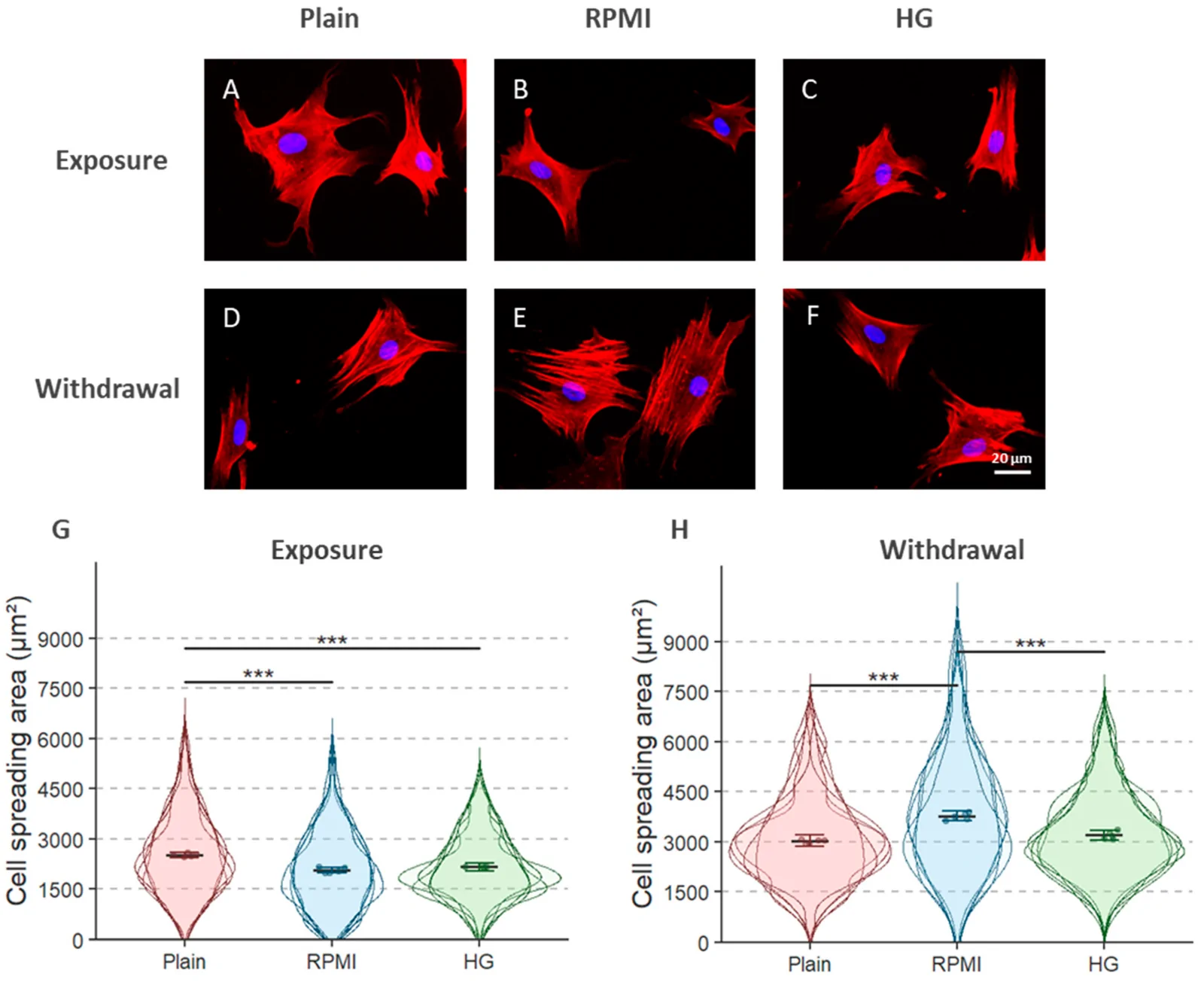
Alterations in AD-MSCs morphology induced by myeloma (RPMI-8226) and leukemia (HG-3) secretomes after 5 h of incubation on collagen-coated surfaces. Fluorescence images of AD-MSCs cultured for 3 days in control medium ((**A**), Plain) or in the presence of RPMI-8226 (RPMI; (**B**)) or HG-3 secretomes (HG; (**C**)) termed “Exposure” Cells treated under the same conditions, followed by an additional 3-day culture in secretome-free medium (“Withdrawal”; (**D**–**F**)) to assess the persistence of secretome-induced effects. Cells were stained for actin (red) and nuclei (blue). Diagrams show the corresponding distribution of cell spreading areas for “Exposure” (**G**) and “Withdrawal” (**H**) culture protocols. Quantitative data are presented as violin plots, with individual cell measurements from each of the five independent replicates shown as separate violins and replicates stacked within each experimental condition. Estimated marginal means (EMM) with 95% confidence intervals (CI) are shown for each condition (*n* = 5 replicates). Model assumptions were assessed using Q-Q plots of model residuals. Data were analyzed using a linear mixed-effects model, followed by pairwise comparisons of EMMs with Tukey adjustment for multiple testing. Asterisks indicate statistical significance (*p* ≤ 0.001, ***). Scale bar: 20 µm.

As individual MSC populations exhibit substantial variability in morphology, growth kinetics, and functional output, we used CellProfiler-based quantitative analysis to obtain an objective assessment of these parameters. As shown in Figure 2, upon adhesion to collagen, both RPMI-8226 and HG-3 cancer cell secretomes alter the overall morphology of stem cells, particularly their size (Figure 2B,C) consistent with the significant reduction in cell spreading area (Figure 2G). For both RPMI-8226 and HG-3, the spreading area decreased to 2068 ± 45 and 2168 ± 56 µm^2^, which was statistically significant compared with control cells (2521 ± 40 µm^2^, *p* ≤ 0.001) (Figure 2A).

Conversely, when the same samples were cultured for an additional 3 days in secretome-free conditions, the post-withdrawal effects diverge markedly (Figure 2G,H). HG-3-treated cells showed a distinct recovery in the spreading area (2163 ± 56 → 3002 ± 71 µm^2^), with no significant differences compared to the control (Figure 2D,F) and consistent with a reversible morphological adaptation. In contrast, RPMI-8226-treated cells (Figure 2E) exhibited a clear rebound effect (2068 ± 45 → 3772 ± 72 µm^2^), suggesting that the induced morphological expansion still manifests after removal of the secretome.

The observed decrease in spreading area under RPMI-8226 and HG-3 “Exposure” indicates that tumor secretomes interfere with both attachment and subsequent cytoskeletal expansion (described in Section 2.5), while the divergent rebound after “Withdrawal” reflects how each secretome imprints a different collagen response.

#### 3.1.2. Cell Proliferation Assay and Senescence-Associated Activity

Cell proliferation was evaluated morphologically via counting the cells per field at 24, 48 and 72 h under both “Exposure” and “Withdrawal” conditions as defined above.

**Figure 3:**
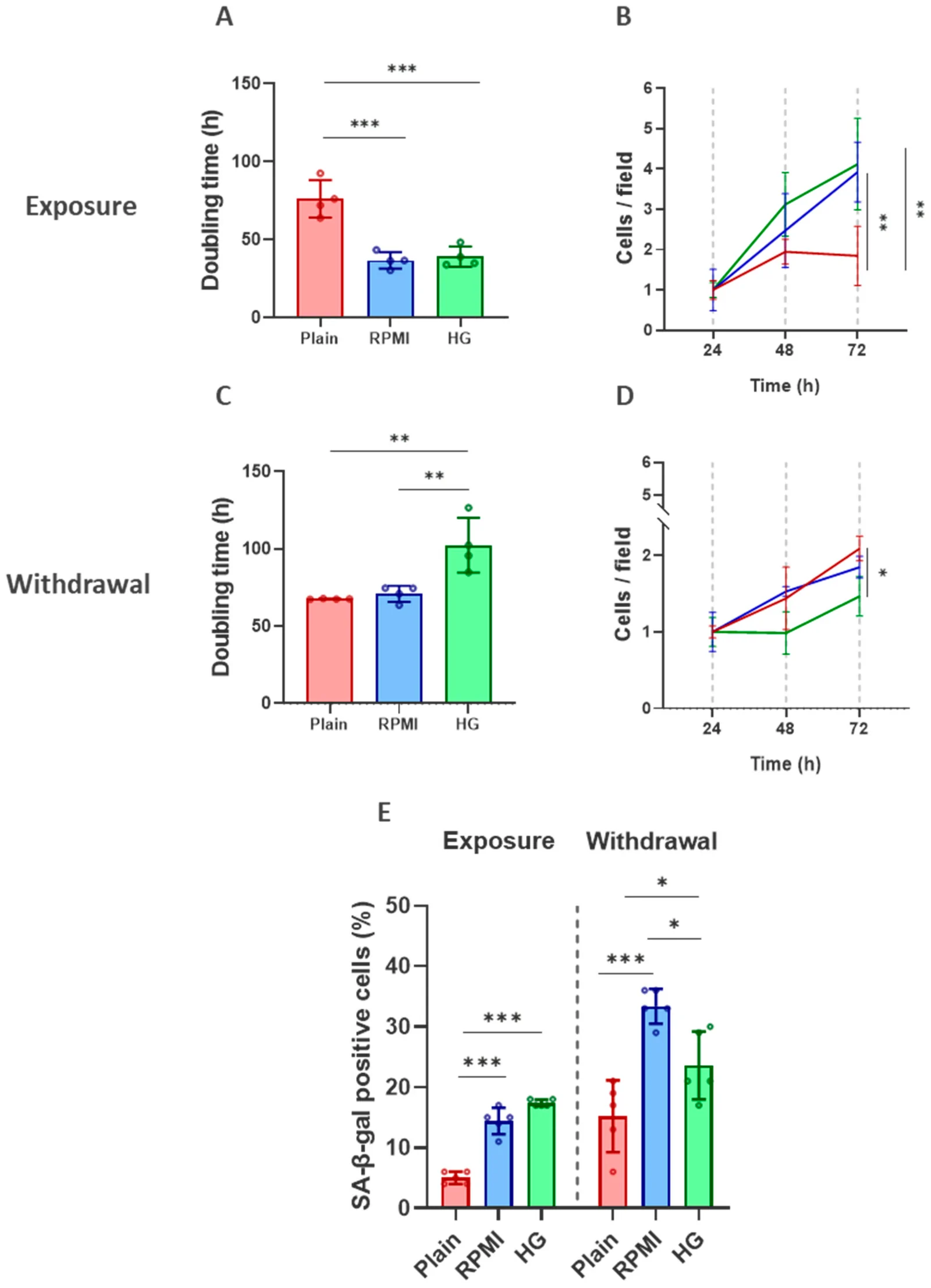
Cancer cell secretomes from myeloma (RPMI-8226) and leukemia (HG-3) cell lines promote accelerated proliferation and β-galactosidase activity in AD-MSCs. Cells were analyzed after 72 h of secretome exposure (“Exposure”; (**A**,**B**)) and after an additional 72 h of subculture in secretome-free medium (“Withdrawal”; (**C**,**D**)). Samples treated with RPMI-8226 secretome are shown in blue (RPMI), whereas those treated with HG-3 secretome are shown in green (HG). AD-MSCs maintained in control medium are depicted in red and labeled as “Plain.” Cell doubling time was calculated over the full 72-h period (**A**,**C**), while the corresponding proliferation curves (**B**,**D**) present normalized cell density per image, with statistical comparisons performed at the 72-h time point. (**E**) SA-β-gal positive cells (%, at the 72-h time point) are shown in panel E again under both “Exposure” and “Withdrawal” conditions. Data are presented as mean ± SD (*n* = 5). Statistical significance was determined using one-way ANOVA with Tukey’s multiple comparisons test. Asterisks indicate statistical significance: *p* ≤ 0.05 (*), *p* ≤ 0.01 (**), *p* ≤ 0.001 (***).

The data in Figure 3A show that AD-MSCs exhibit a markedly reduced cell-doubling time compared with untreated controls when exposed to secretomes from either RPMI-8226 or HG-3 cells under the “Exposure” condition. This accelerated proliferation is further reflected in Figure 3B, where AD-MSCs more than double their proliferation rate relative to control cells.

However, when AD-MSCs exposed to secretomes are subsequently cultured in secretome-free medium (“Withdrawal” conditions), their behavior diverges depending on the source of the secretome. Cells pre-treated with RPMI-8226 secretome largely recover their proliferative capacity, returning to levels close to the control (Plain). In contrast, cells pre-treated with HG-3 secretome exhibit a significantly increased doubling time (Figure 3C), accompanied by a sustained reduction in proliferation across all measured time points (Figure 3D). This pattern indicates a clear “Withdrawal” effect specifically associated with HG-3 secretome exposure.

Under direct (“Exposure”) treatment, SA-β-gal levels are already significantly elevated compared with controls. Under “Withdrawal” conditions, both retain their marked increase in SA-β-gal positive cells, however, RPMI-treated cells present a significantly higher senescence-related activity (*p* ≤ 0.01) compared to both the Plain and HG conditions (Figure 3E). These findings suggest that the age-related changes are not the result of acute cytotoxicity from the secretomes. Instead, it appears linked to a longer-term functional reprogramming of AD-MSCs toward a cancer-associated-like phenotype.

#### 3.1.3. Effect of Cancer Cell Secretomes on Cell Cycle Progression

**Figure 4:**
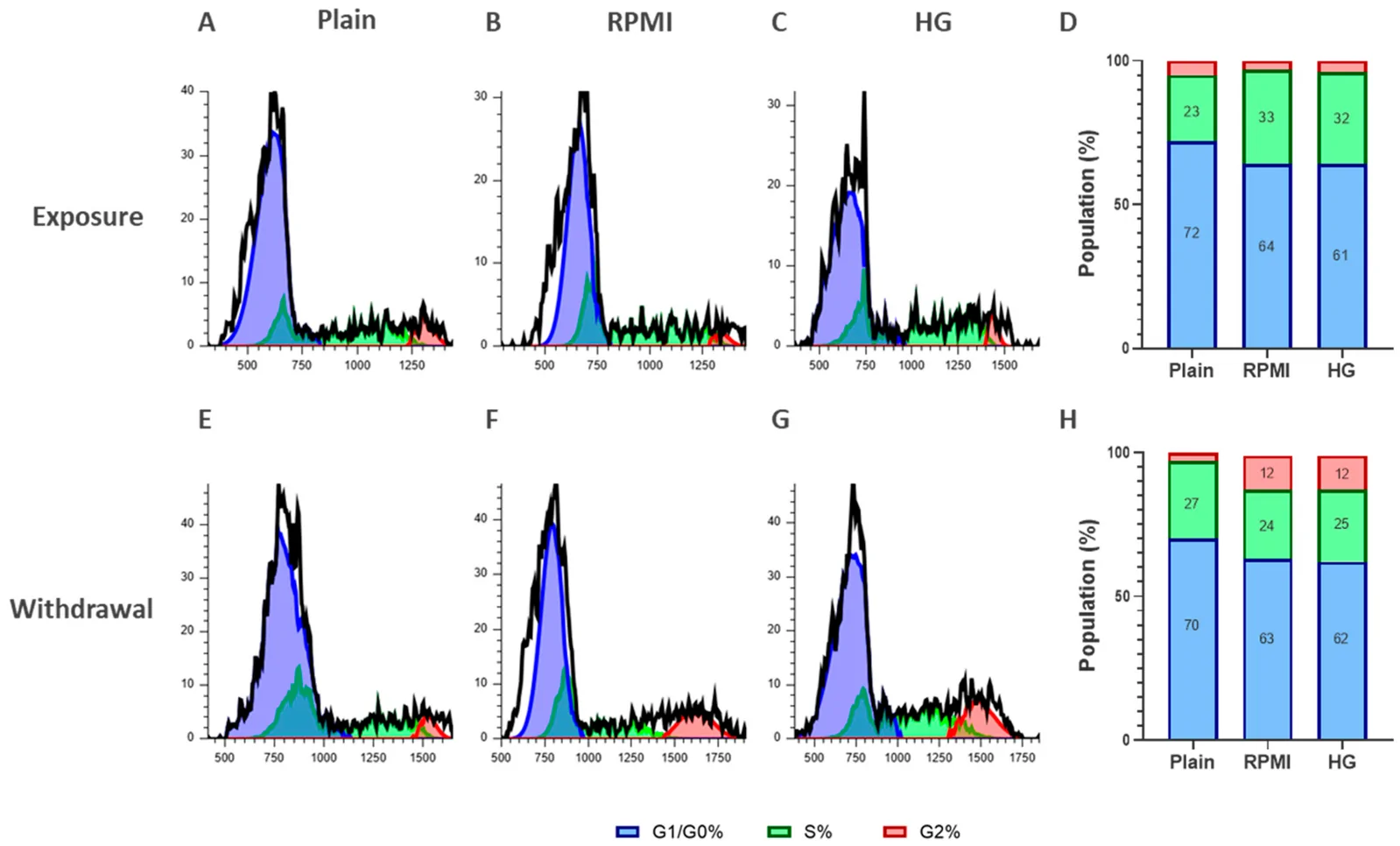
Cancer cell secretome selectively modulates cell cycle distribution in AD-MSCs. Fluorescence-based image cytometry analysis of nuclear DNA content in Hoechst-stained AD-MSCs cultured for 72 h with cancer-cell secretome (“Exposure”), followed by an additional 72 h in normal medium after secretome removal (“Withdrawal”). The experiment includes three conditions: standard medium with 10% FBS (plain), medium supplemented with RPMI-8226 secretome (RPMI), and medium supplemented with HG-3 secretome (HG). (**A**–**C**,**E**–**G**) display representative DNA histograms, where event count (y-axis) is plotted against total nuclear fluorescence intensity (x-axis), reflecting DNA content. Cell cycle phases are color-coded: G0/G1 (blue), S (green), and G2/M (red). AD-MSCs fluorescence images were analyzed after the end of each time point (72 h after secretome addition and 72 h after secretome removal). (**D**,**H**) presents quantitative analysis of cell cycle phase distribution, expressed as a percentage of the total cell population for AD-MSCs in “Exposure” (**upper row**) and “Withdrawal” (**lower row**) conditions. Color coding is consistent across the two panels.

To investigate the effects of cancer cell secretome on cell-cycle dynamics, fluorescence-based image cytometry analysis of DNA content was performed across “Exposure” and “Withdrawal” states (Figure 4). Across all samples, the majority of cells were distributed within the G0/G1 phase, characterized by a dominant peak in the lower fluorescence intensity range. However, clear condition-dependent alterations in cell-cycle progression were observed.

In the “Exposure” group, cells cultured under control conditions (Plain) exhibited a typical proliferative profile, with a predominant G0/G1 population and minimal accumulation in S or G2/M phases. In contrast, RPMI and HG conditions induced a modest expansion of the S population, suggesting enhanced cell-cycle entry and DNA synthesis activity. Taken together with the reduced doubling time, these data support am initial shift toward a more replication-focused proliferative phenotype.

More pronounced effects were observed in the “Withdrawal” group. While plain samples showed only minor changes compared to their induced counterparts, “Withdrawal” RPMI demonstrated a clear rightward shift in DNA content distribution, indicative of increased accumulation in the G2/M phase. Most notably, the “Withdrawal” HG condition showed an apparent alteration in cell-cycle distribution, characterized by an expansion of the G2/M population and a relative reduction in the G0/G1 fraction. Together with the observed reduction in proliferation, this pattern is consistent with delayed cell-cycle progression or G2/M arrest.

Collectively, these results indicate that tumor-associated transitions, particularly under “Withdrawal” conditions, promote accumulation of cells in the G2/M phase, suggesting activation of cell-cycle checkpoint mechanisms that restrict progression into mitosis.

#### 3.1.4. Live/Dead Assay

To further rule out the possibility that cancer cell-derived secretomes exert direct cytotoxic effects, which is particularly imaginable under direct exposure (“Exposure”) conditions, we performed a viability (live/dead) assay at 24 and 72 h. The assay was conducted under both “Exposure” and “Withdrawal” conditions.

**Figure 5:**
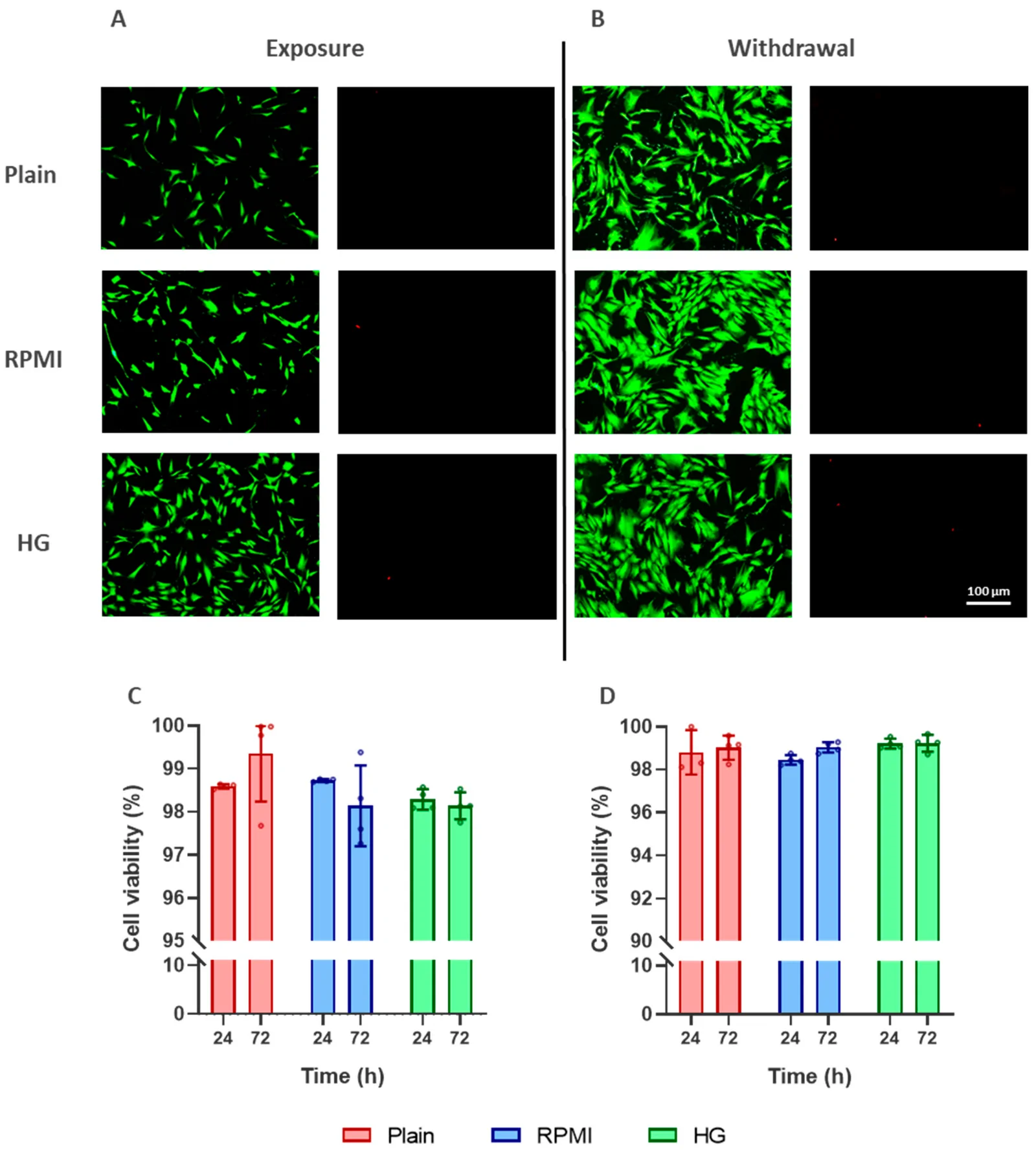
AD-MSCs maintain high viability when exposed to cancer cell secretomes from myeloma (RPMI-8226) and leukemia (HG-3) cell lines. Fluorescent live/dead images are shown for all experimental conditions (Plain, RPMI-8226-treated, and HG-3-treated) under both “Exposure” (**A**) and “Withdrawal” (**B**) settings. Images were captured at 10× magnification at the 72-h time point with the left panel showing viable Calcein AM-positive cells (green) and the right panel showing dead propidium iodide-positive cells (red). Quantitative analyses of AD-MSC viability is presented under “Exposure” (**C**) and “Withdrawal” (**D**) conditions at 24- and 72-h time points, respectively. Data are shown as mean ± SD (*n* = 4). Error bars exceeding 100% were truncated at the upper limit of the scale. Statistical significance was assessed using one-way ANOVA followed by Tukey’s multiple comparisons test. Scale bar: 100 µm.

As seen in Figure 5, across all treatment settings and time points, AD-MSC viability remained consistently high (99–100%), with most cells stained in green and only sparse appearance of red-colored nuclei (Figure 5A,B right rows) with no significant differences compared to controls (Figure 5C,D). These findings confirm the absence of both “Exposure” and “Withdrawal” cytotoxicity from the cancer cell secretomes.

### 3.2. Effect of Cancer-Derived Secretomes on Collagen Remodeling

The primary aim of this study is to define how cancer-cell secretomes shape MSC behavior, focusing on their interaction with and remodeling of type I collagen, a widely distributed fibrillary component of the ECM. However, we first evaluated the direct effects of cancer cells on collagen during adhesion. Defining this baseline allows us to distinguish and interpret effects that require direct physical contact with cancer cells from those mediated exclusively by their secreted paracrine factors.

#### 3.2.1. Direct Interaction of Lymphoma and Myeloma Cells with Collagen

**Figure 6:**
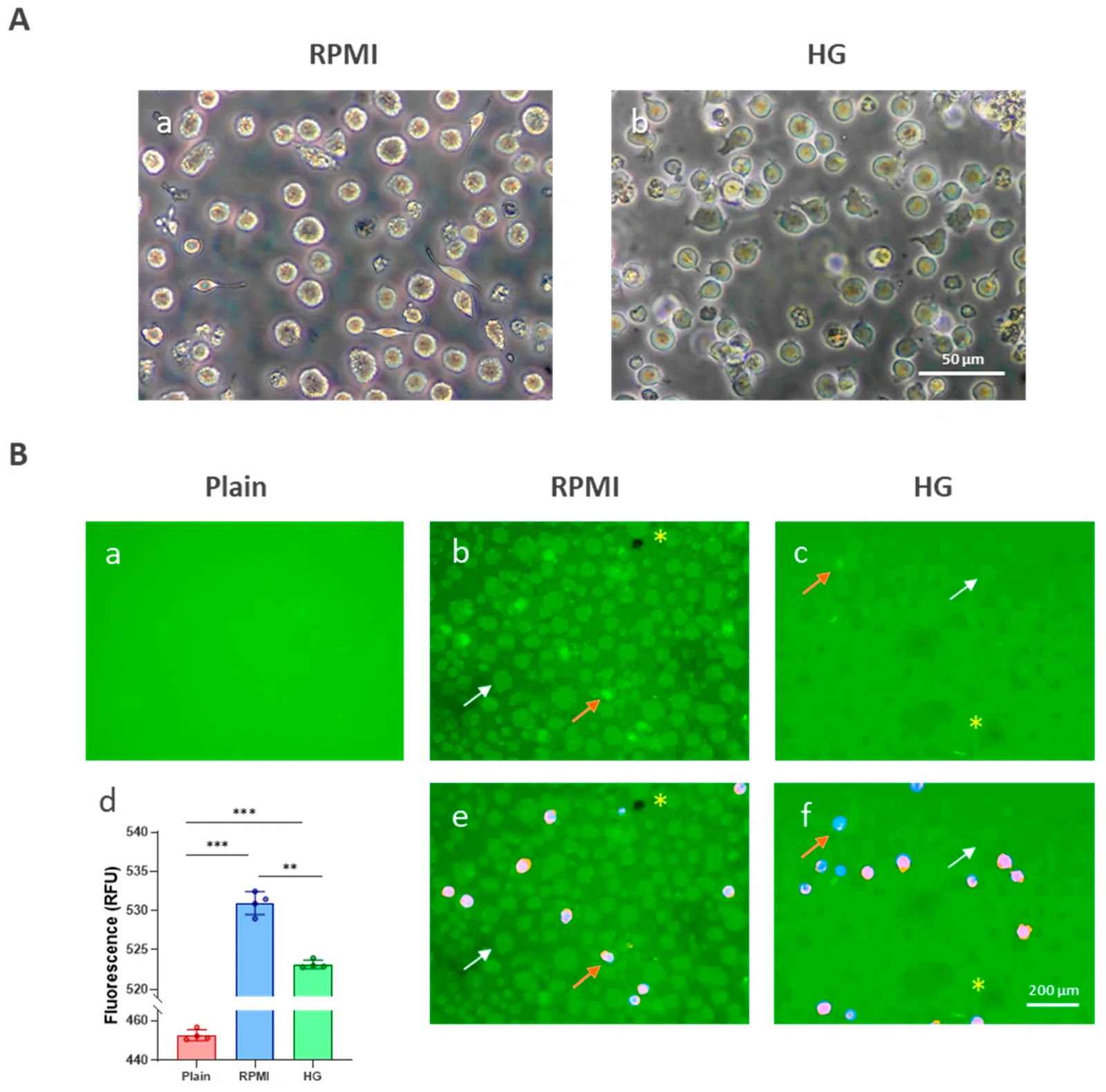
Direct interaction of lymphoma and myeloma cells with collagen establishes a baseline for distinguishing contact-dependent from paracrine effects. (**A**) Representative phase-contrast images of RPMI-8226 (**a**) and HG-3 cells (**b**), illustrating their ability to form transient adhesive contacts with tissue-culture plastic despite being predominantly suspension-growing cell lines. Images were acquired directly from culture flasks at 20× magnification. (**B**) Cancer cells were allowed to attach for 2 h to FITC-collagen-coated substrate in serum-free medium. The associated collagen remodeling (green) is presented (**a**–**c**), and merged images with actin (red) and nuclei (blue) are also shown (**e**,**f**). Image (**a**) is the plain substrate, (**b**,**e**)—RPMI-8226, and (**c**,**f**)—HG-3 remodeling. Orange arrows indicate cell attachment locations corresponding with higher substrate fluorescent intensity. White arrows indicate places where cells have transiently adhered and were subsequently washed away, leaving a visible remodeled region. Yellow asterisks denote diffuse, darker “shadow-like” regions in the substrate. (**d**) Fluorescence measurements of the collected supernatants demonstrate increased release of FITC-collagen fragments in the presence of cancer cells compared with collagen-only (Plain) controls. Data are presented as relative fluorescence units (RFU) mean ± SD (*n* = 4). Statistical significance was assessed using one-way ANOVA, followed by Tukey’s multiple comparisons test. Asterisks denote statistical significance of *p* ≤  0.01 (**), and *p*  ≤  0.001 (***). Scale bar: 50 µm (Panel (**A**)); 200 µm (Panel (**B**)).

To address this, we assessed the collagen remodeling activity of RPMI-8226 mye-loma and HG-3 lymphoma cells. Although these cells are typically weakly adhesive and therefore easily maintained in suspension, as shown in Figure 6A, images (a) and (b), respectively, both types of cells readily formed limited adhesive contacts even with tissue-culture plastic, presumably using some adsorbed serum-derived proteins.

To model the specific interaction of cancer cells and also MSCs with a collagen matrix, we used FITC collagen-coated substrata. The FITC-labeled collagen used in these assays exhibits partial fluorescence quenching due to the high local density of fluorophores along the collagen fibrils. Such closely spaced FITC molecules undergo mutual short-range energy transfer, resulting in self-quenching consistent with established Förster (FRET-mediated) quenching mechanisms [50]. As shown in Figure 6B, once attached, the cancer cells induced pronounced enzymatic remodeling of the underlying collagen substrate, evidenced by marked local de-quenching and sharply increased green fluorescence beneath the cells (yellow arrow), with their positions confirmed in the merged actin/nucleus images (Figure 6(Be,Bf)). In addition to these bright remodeling zones, numerous fainter, cell-shaped patterns were visible, presumably corresponding to regions where cells had transiently adhered and were subsequently washed away, leaving behind areas of moderately de-quenched collagen (white arrows). This remodeling activity was more pronounced in the RPMI-8226 samples (Figure 6(Bb,Be)), although discrete de-quenching spots were also detectable beneath several HG-3 cells (Figure 6(Bc,Bf)). In both cell types, diffuse darker “shadow-like” regions of FITC-collagen degradation marked with (*), not associated with any current cell body, were also observed. Consistent with these observations are the fluorescence measurements in the culture supernatant showing a significantly increased FITC fluorescence signal in the presence of cells compared with the spontaneous release from substrata alone (Figure 6(Bd), *p* ≤  0.001), indicating a significantly increased release of fluorescent collagen fragments into the medium.

#### 3.2.2. Differences in Cytokine Secretion by the Tumor Cells

**Figure 7:**
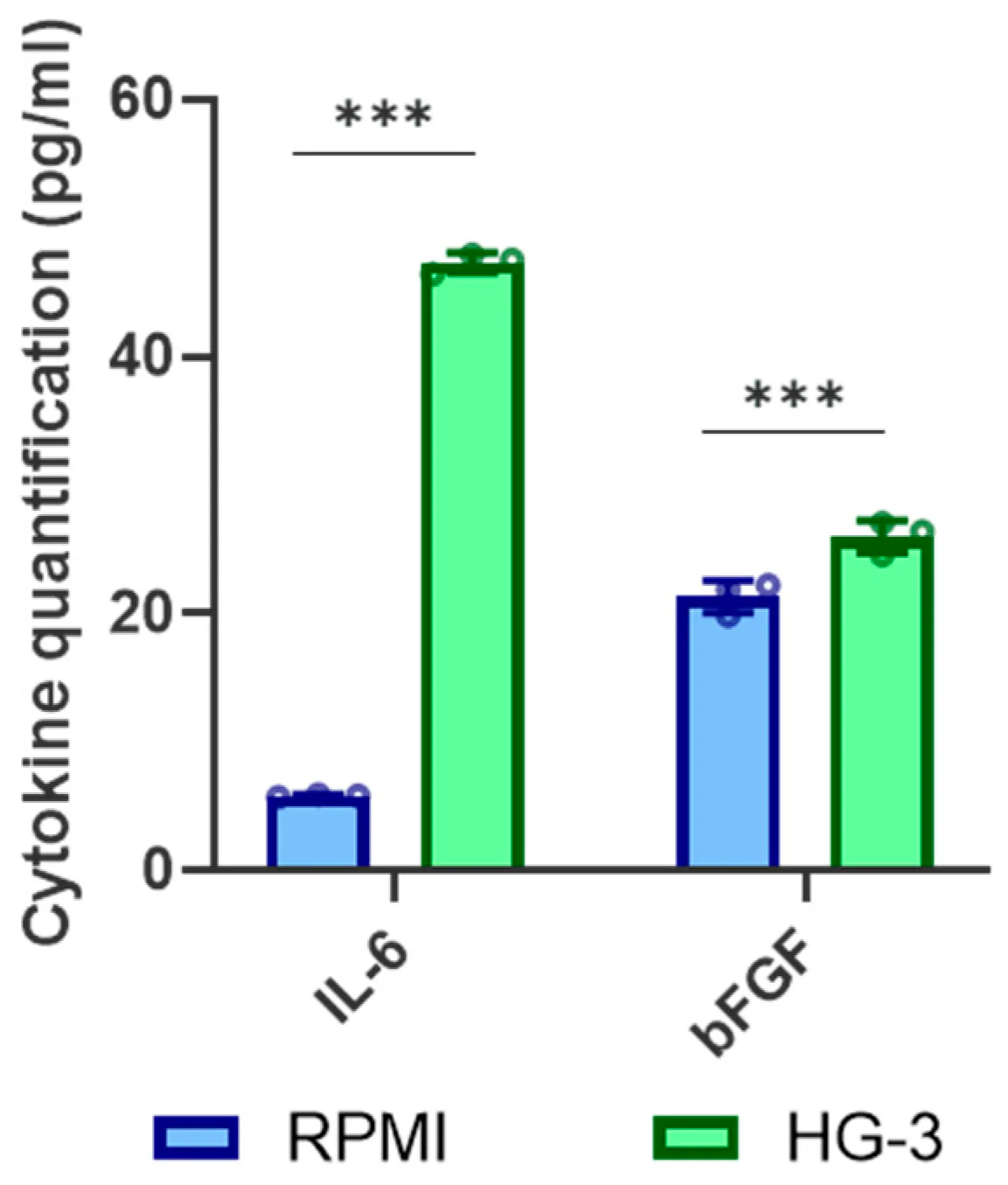
Differential secretion of IL-6 and bFGF between lymphoma and myeloma cell secretomes, measured by ELISA. Quantitative data are presented as mean ± SD (*n* = 3). Statistical analysis was performed using one-way ANOVA followed by Tukey’s multiple comparisons test. Asterisks indicate statistical significance at *p* ≤ 0.001 (***).

Given the subsequent analyses of cancer-cell secretome signaling and its impact on MSC collagen-remodeling activity, we also quantified the levels of the stromal-modulating cytokines IL-6 and bFGF in the conditioned media (Figure 7).

Both IL-6 and bFGF levels were significantly higher in the HG-3-derived secretome than in the RPMI-8226-derived secretome (*p* ≤  0.001). IL-6 levels were approximately 8-fold higher (*p* ≤  0.001), whereas the difference in bFGF levels was less pronounced despite reaching statistical significance. Nevertheless, comprehensive proteomic analysis and vesicle-specific profiling would be required to identify the individual secretome components contributing to these effects and represents an important direction for future studies.

#### 3.2.3. Effect of Cancer Cells Secretomes on Stem Cells Collagen Remodeling

**Figure 8:**
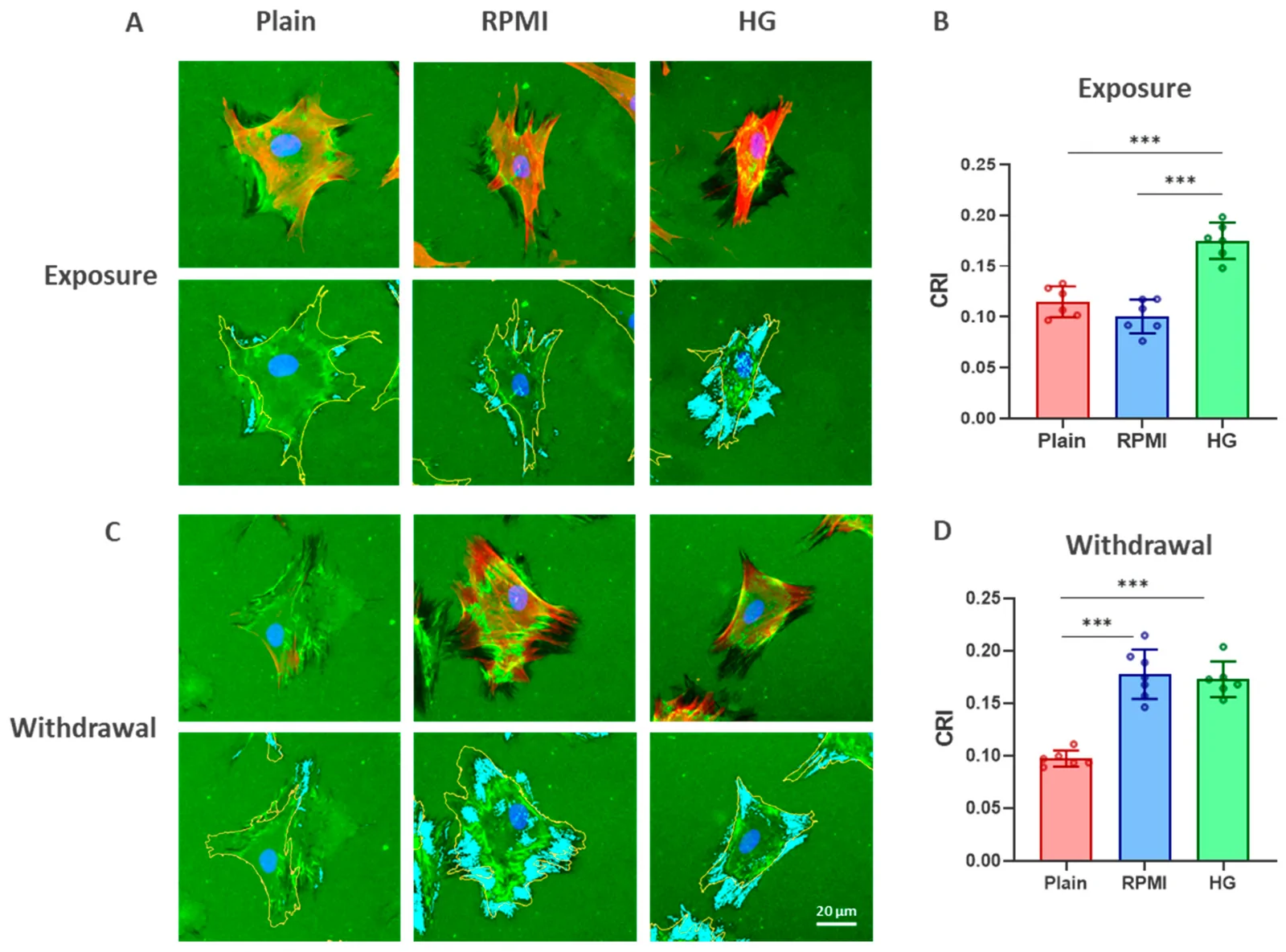
FITC-collagen remodeling by AD-MSCs after exposure to cancer cell secretomes from myeloma (RPMI-8226) and leukemia (HG-3) cell lines. Representative fluorescence micrographs of AD-MSCs cultured for 5 h on FITC-collagen substrates under control conditions or after treatment with RPMI-8226 or HG-3-derived secretomes. Cells were analyzed under both “Exposure” (**A**) and “Withdrawal” (**C**) protocols. To visualize cell morphology, fixed samples were stained for actin (red) and nuclei (blue), overlaid on the fluorescent background of adsorbed FITC-collagen (green) (upper panels in (**A**,**C**)). CellProfiler software was used to identify remodeled collagen regions (light blue) and superimpose them onto cell boundaries (orange outline) to quantify the collagen removal index (CRI). Quantification of collagen remodeling is shown for “Exposure” (**B**) and “Withdrawal” (**D**) conditions. Data are presented as mean ± SD (*n* = 6). Statistical analysis was performed using one-way ANOVA followed by Tukey’s multiple comparisons test. Asterisks indicate statistical significance: *p* ≤ 0.001 (***). Scale bar for panels (**A**,**C**): 20 μm.

When AD-MSCs were cultured under “Exposure” conditions (Figure 8A), the presence of HG-3 secretome resulted in markedly greater collagen substrate remodeling compared with both the Plain control and RPMI-8226-treated cells. This was also reflected in the normalized images of this enhanced matrix-remodeling activity, further confirmed by morphometric analysis of FITC-collagen degradation patterns, showing a statistically significant increase in remodeled area (Figure 8B, *p* ≤  0.001). To further account for differences in cell spreading, remodeling activity was normalized to cell area and expressed as the remodeled collagen area-to-cell-spreading area ratio, introduced here as the collagen removal index (CRI). This metric indicates that HG-3-treated cells exhibit increased remodeling activity relative to both RPMI-8226-treated and control cells, suggesting that the effect reflects active ECM-interacting capacity rather than purely morphological changes.

In contrast, under “Withdrawal” conditions, both HG-3- and RPMI-derived secretomes induced substantially higher levels of collagen reorganization relative to control cells (Figure 8C).

CRI-based analysis confirmed that both treatments maintained significantly elevated remodeling efficiency compared to controls (Figure 8D, *p* ≤ 0.001), suggesting a partially persistent activation of ECM-modifying programs following secretome withdrawal.

Cell spreading area and remodeled collagen area were quantified on an image basis and normalized to cell number. No significant differences were observed between the Plain, RPMI and HG conditions, likely due to the smaller statistical sample size. On the other hand, the absolute remodeled collagen area (Table 1) alone did not fully capture the differences in collagen-remodeling phenotypes observed in Figure 8 and Figure S2. In contrast, normalization of the remodeled collagen area to cell spreading area using the CRI more closely reflected the remodeling phenotype observed in the fluorescence images. This indicates that the differences in CRI are not solely attributable to cell spreading area but rather to remodeling area.

The comparison between “Exposure” and “Withdrawal” conditions revealed distinct response dynamics for both secretomes. HG-3-treated cells maintained nearly unchanged CRI values (0.18 ± 0.02 vs. 0.17 ± 0.02), indicating a stable collagen remodeling phenotype that persisted regardless of interrupted stimulation. In contrast, RPMI-8226-treated cells exhibited an increase in CRI following secretome withdrawal (from 0.10 ± 0.02 to 0.18 ± 0.02), indicating that maximal remodeling efficiency emerges after the removal of the stimulus.

These findings indicate that the HG-3 secretome promotes an immediate and sustained activation of collagen remodeling, whereas the RPMI-8226 secretome induces a delayed, adaptive response characterized by post-stimulus functional enhancement. This difference indicates distinct modes of cell programming by leukemia- versus myeloma-derived paracrine signals.

**Figure 9:**
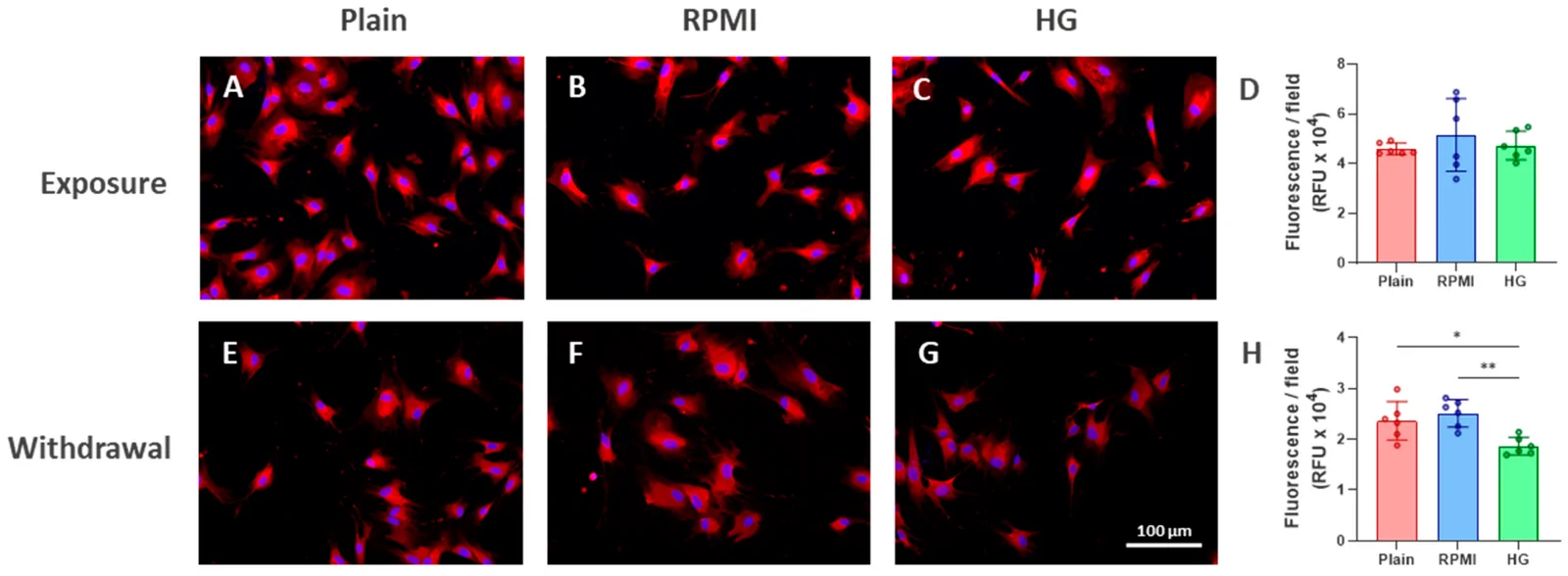
Cancer cell secretome differentially regulates collagen synthesis by AD-MSCs. Immunofluorescence imaging and quantitative analysis of human collagen type I content in AD-MSCs cultured on rat collagen type I substratum after 5 h of incubation. The cells were seeded on rat collagen substrata after being cultured under three conditions: (**A**,**E**) control (Plain), (**B**,**F**) treated with RPMI-8226-derived secretome (RPMI) and (**C**,**G**) with HG-3-derived secretome (HG). Representative images of cells stained for nuclei (Hoechst, blue) and collagen type I (Alexa Fluor 555, red) under (**A**–**C**) “Exposure” and (**E**–**G**) under corresponding “Withdrawal” conditions. Quantification of intracellular collagen fluorescence normalized to cell number is presented in (**D**,**H**), corresponding to “Exposure” and “Withdrawal” conditions. Cell boundaries are defined using CellProfiler based on phalloidin-stained actin. Statistical analysis was performed using two-way ANOVA, followed by Tukey’s multiple comparisons test. Significance is indicated as follows: *p* ≤ 0.01 (*) and *p* ≤ 0.01 (**). Scale bar: 100 μm.

Further quantitative analysis of collagen type I cellular content revealed that AD-MSCs maintained comparable levels of collagen production across most conditions (Figure 9) under “Exposure” condition effects.

Fluorescence intensity measurements based on the images (Figure 9A–C) and normalized to cell number (Figure 9D) showed no significant differences relative to control cells, indicating that short-term exposure to tumor-derived secretomes does not alter the quantity of intracellular collagen. However, under “Withdrawal” conditions after HG-3 treatment, a significant reduction in collagen synthesis was observed (Figure 9G,H) that was not evident during the “Exposure” phase. Since the myeloma cell-treated samples exhibit stable collagen synthesis in all conditions, it suggests a cell-type-specific response.

### 3.3. Secretome-Induced Elevation of Key Cytokines in the Conditioned Medium of MSCs

**Figure 10:**
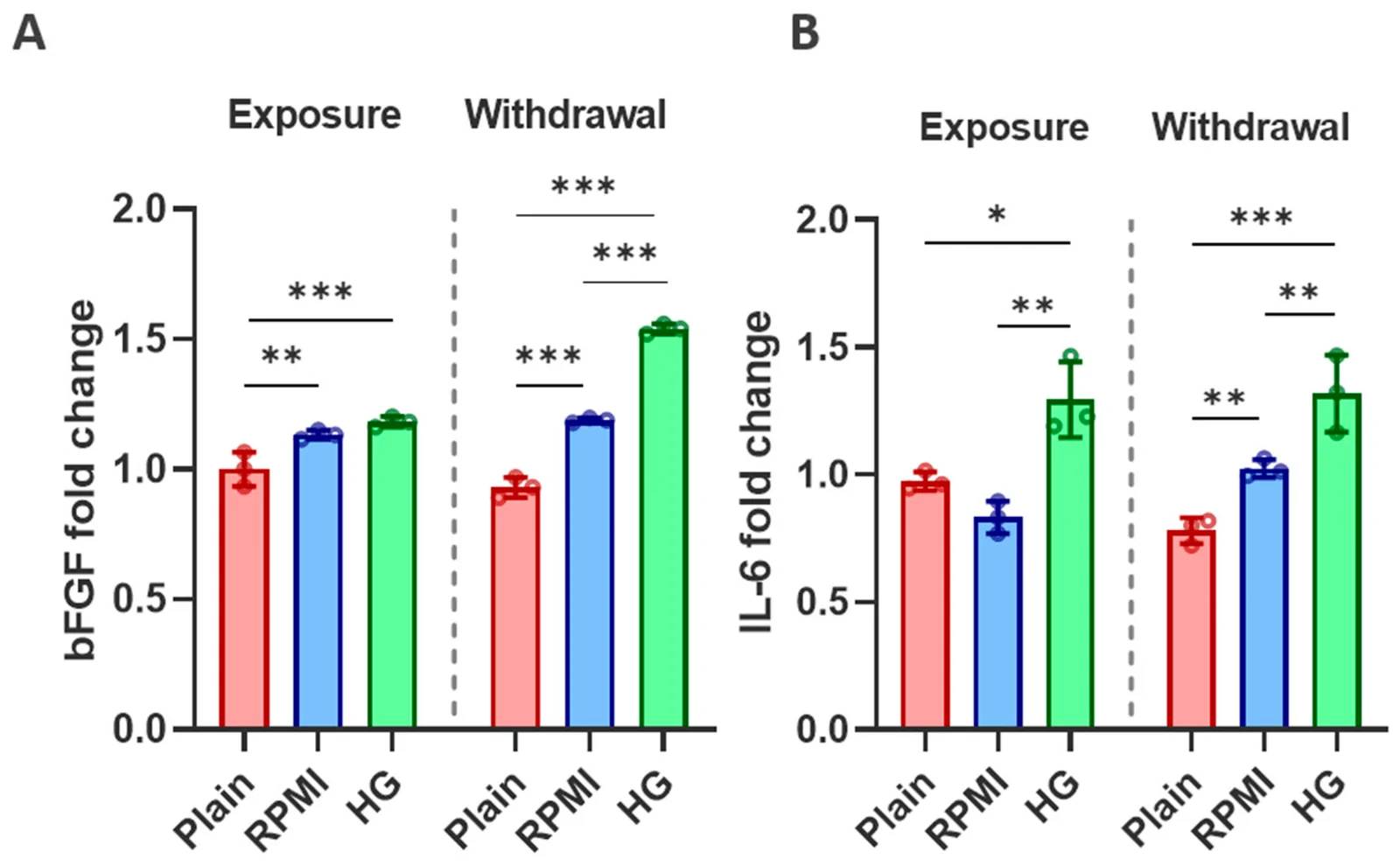
Cancer cell secretomes elevate bFGF (**A**) and IL-6 (**B**) production in MSCs. Cytokine concentrations were measured under three experimental conditions: untreated control (Plain), RPMI-8226 secretome–treated (RPMI), and HG-3 secretome–treated (HG) MSCs. The bar graphs show cytokine levels in both the “Exposure” and “Withdrawal” settings, reflecting MSC secretory responses to myeloma- and leukemia-derived secretomes. Data are presented as fold change relative to the control and expressed as mean ± SD (*n* = 3). Statistical significance was determined using one-way ANOVA with Tukey’s multiple comparisons test. Asterisks indicate statistical significance: *p* ≤ 0.05 (*), *p* ≤ 0.01 (**), *p* ≤ 0.001 (***).

To confirm the putative cancer-associated changes of MSCs in the presence of RPMI-8226 and HG-3 secretomes, we examined the potentially elevated levels of key stromal-modulating cytokines, bFGF and IL-6, in the conditioned media collected during both the early “Exposure” phase and in later “Withdrawal” conditions of MSC culture (Figure 10).

In the early “Exposure” phase, we observed a significant (*p* ≤ 0.001) increase in bFGF in the MSC supernatant in the presence of both RPMI-8226 and HG-3 secretomes, whereas IL-6 was elevated only in the HG-3 setting. During the subsequent “Withdrawal” phase, this cytokine upregulation persisted, and notably, IL-6 also became elevated in the RPMI-8226 condition, suggesting a delayed response of MSCs.

### 3.4. Tumor-Cell Secretomes Enhance MSC Motility

To assess the motility response of AD-MSCs, we employed the classical scratch assay. AD-MSCs were maintained under the “Exposure” and “Withdrawal” conditions described above. Following scratch formation, cells were monitored for up to 24 h in a humidified chamber of an inverted microscope using phase-contrast time-lapse imaging.

**Figure 11:**
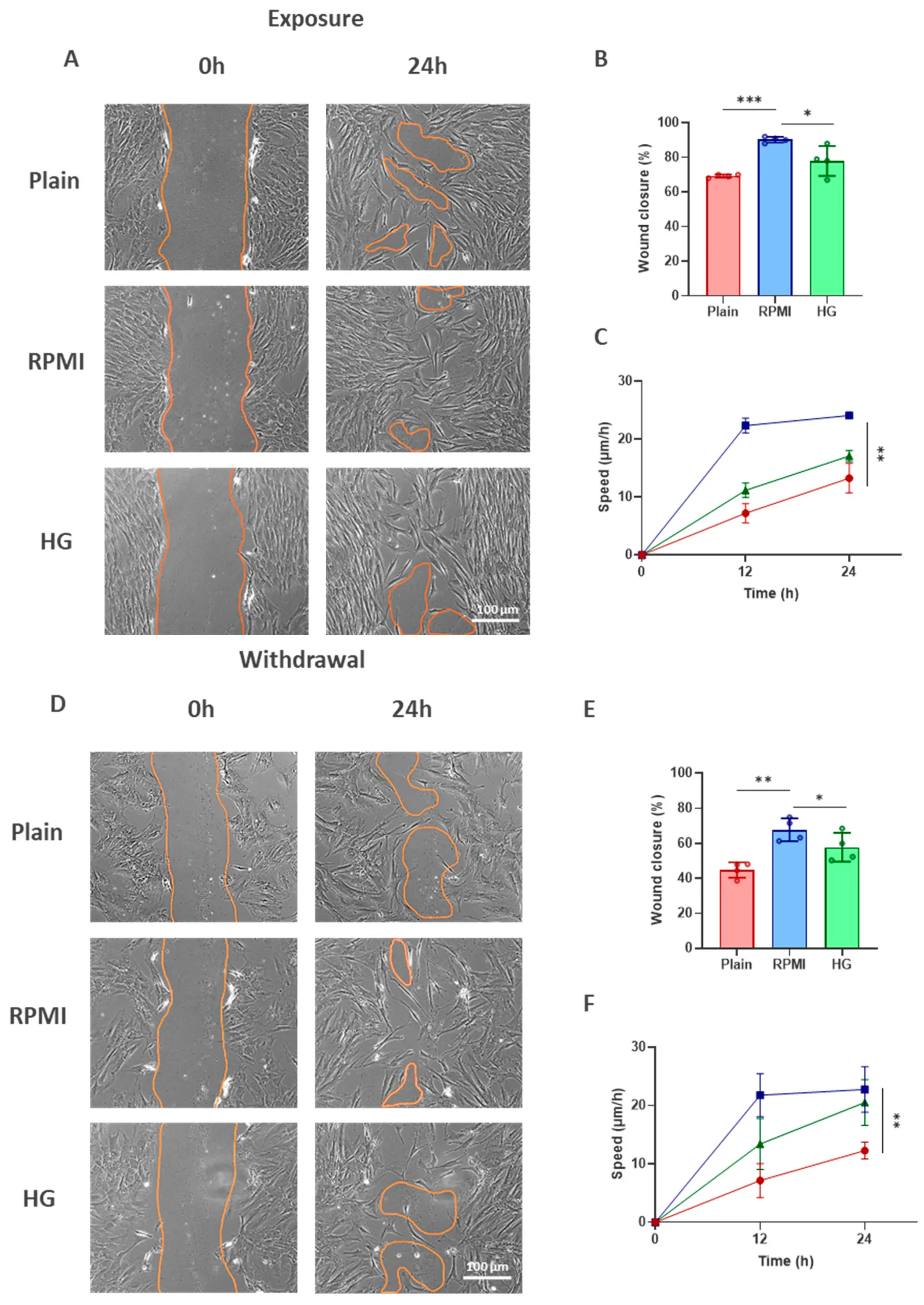
AD-MSC motility accelerates after treatment with cancer cell secretomes from myeloma (RPMI-8226) and leukemia (HG-3) cell lines. Representative phase-contrast images from wound-healing assays performed under “Exposure” (**A**) and “Withdrawal” (**D**) conditions. An orange line marks the initial wound area as well as the closed region at the endpoint. “Plain” denotes untreated control cells, while “RPMI” and “HG” indicate treatment with the corresponding tumor cell-derived secretomes. The presented images were captured at 0 h and 24 h. Graphs (**B**,**E**) show the percentage of wound closure at the 24-h time point. Graphs (**C**,**F**) depict changes in cell population motility speed over time (0, 12, and 24 h), analyzed up to the experimental endpoint. Data are presented as mean ± SD (*n* = 4). Statistical analysis was performed using one-way ANOVA followed by Tukey’s multiple comparisons test. Asterisks indicate statistical significance at *p* ≤ 0.05 (*), *p* ≤ 0.01 (**), *p* ≤ 0.001 (***). Scale bar: 100 µm.

As shown in Figure 11, AD-MSCs exhibited a marked increase in both wound-closure speed and overall closure extent under both “Exposure” and “Withdrawal” conditions compared with control cells (Figure 11A–F). On the left side are shown the real images (at time 0 and 24 h) for “Exposure” and “Withdrawal” samples (Figure 11A and Figure 11D, respectively), while on the right are the corresponding quantifications (Figure 11B,C,E,F). Notably, the RPMI secretome elicited a substantially stronger pro-migratory effect in both conditions, nearly twice that of the control, whereas HG-3 secretome exposure produced a more moderate increase during the “Exposure” phase (Figure 11B,C) that became significantly enhanced in the “Withdrawal” settings (Figure 11E,F, *p* ≤ 0.001).

## 4. Discussion

The present study provides an integrated functional analysis of how cancer cell-derived secretomes from two B-cell malignancies, RPMI-8226 (multiple myeloma (MM)-derived) and HG-3 (chronic lymphocytic leukemia (CLL)-derived), modulate the early behavior of adipose derived mesenchymal stromal cells (AD-MSCs). Although MM and CLL arise from different B-cell lineages and occupy distinct anatomical niches, both malignancies rely heavily on stromal interactions, chemokine signaling, and extracellular matrix (ECM) remodeling for homing, retention, and disease progression [51,52]. This shared microenvironmental dependence provides a strong rationale for examining their secretomes in parallel. Our findings reveal both common stromal responses and lineage-specific differences, offering insight into how hematologic cancer–derived soluble cues can reprogram MSCs outside their native niche [6,53].

### 4.1. Shared Stromal Responses to MM and CLL Secretomes

Despite their biological differences, both secretomes induced a coordinated early response in AD-MSCs, including reduced spreading area, altered morphology, accelerated proliferation during direct exposure, increased senescence-associated β-gal activity, enhanced motility, and elevated collagen remodeling. These shared effects suggest that AD-MSCs exhibit a generic stromal activation program when exposed to malignant B-cell secretomes. The convergence of responses is notable given that MM typically resides in a collagen-rich bone marrow niche [51], whereas CLL traffics dynamically between blood, marrow, and lymphoid tissues [54]. The fact that AD-MSCs, cells normally residing in adipose tissue, respond robustly to both secretomes underscores the potency of tumor-derived paracrine signals and highlights the systemic relevance of stromal reprogramming in hematologic malignancies.

### 4.2. Distinct Stromal Responses Reveal Lineage-Specific MSC Modulation

Although many responses were shared, several phenotypes diverged between MM and CLL secretomes, reflecting their distinct biological contexts.

#### 4.2.1. Morphology and Spreading

Both secretomes reduced spreading area during the “Exposure” phase, consistent with cytoskeletal reorganization and altered integrin signaling typically observed in tumor-educated MSCs [8,55]. However, RPMI-8226 induced a pronounced post-withdrawal increase in spreading, whereas HG-3 induced only moderate recovery. This suggests that MM secretomes may prime MSCs for delayed cytoskeletal expansion, potentially reflecting the strong ECM-rich niche dependence of myeloma cells.

#### 4.2.2. Proliferation and Senescence

Direct exposure to both secretomes accelerated proliferation, yet the “Withdrawal” responses diverged: RPMI-8226-conditioned MSCs returned to baseline, whereas HG-3-conditioned MSCs exhibited sustained growth reduction. This aligns with reports that cancer secretomes, such as those from CLL and MM, induce inflammatory and SASP-like phenotypes in MSCs [32,56]; moreover, MM secretomes promote stromal expansion and remodeling [57]. The paradoxical combination of increased proliferation and increased senescence-associated activity suggests that accelerated cycling during the “Exposed” phase may trigger replication-associated senescence, particularly in the HG-3 condition.

#### 4.2.3. Cell Cycle Dynamics

During the “Exposure” phase, AD-MSCs showed an accumulation in S phase, which is consistent with the observed increase in proliferation. Later in the “Withdrawal” phase, both secretomes induced G2/M accumulation, indicating checkpoint activation and potential DNA damage-associated stress [58]. The stronger “Withdrawal” effect suggests a form of paracrine-induced cellular memory, consistent with long-lasting stromal reprogramming described in hematologic malignancies [59].

#### 4.2.4. Motility

Both secretomes enhanced MSC motility, but RPMI-8226 elicited nearly twice the effect observed with HG-3. This may reflect the strong chemotactic profile of MM, where CXCL12/CXCR4 and VLA-4/VCAM-1 interactions dominate niche retention and migration [51]. In contrast, CLL relies more heavily on lymphoid stromal cues and immune cell interactions [54], which may explain the more moderate motility response.

As the AD-MSCs were washed and imaged in secretome-free medium, the scratch assay reflects post-exposure migration rather than ongoing secretome-driven movement. Accordingly, the enhanced motility of RPMI-8226–conditioned MSCs indicates a more durable migratory phenotype, whereas HG-3 induces a more moderate yet still sustained response.

### 4.3. Increased Cytokine Secretion in Response to Tumor Secretomes

Our analyses of the conditioned media provide molecular support for the functional alterations induced in MSCs by tumor-cell secretomes. Both RPMI-8226 and HG-3 secretomes increased bFGF and IL-6 levels during the later “Withdrawal” phase, while IL-6 elevation in the early “Exposure” phase was observed only in the HG-3 condition, indicating a delayed IL-6 response in RPMI-8226-treated MSCs. Given that both cytokines were markedly higher in the HG-3–derived secretome compared with RPMI-8226, it is likely that HG-3 secretes a more cytokine-rich stromal-activating milieu. Interestingly, these elevated paracrine factors in the HG-3 secretome align with the functional outcomes observed in MSCs, where HG-3–treated cells across both the exposure and withdrawal phases exhibited stronger secretome-driven effects compared with RPMI-8226.

Furthermore, bFGF and IL-6 are well-established stromal modulators: bFGF promotes cytoskeletal organization, motility, and ECM-related responses, whereas IL-6 drives inflammatory reprogramming, STAT3 activation, and stromal support of tumor progression. Their coordinated elevation during the MSC response aligns with the enhanced collagen reorganization and increased motility, further supporting the early stromal-modifying program triggered by malignant B-cell secretomes.

### 4.4. Collagen Remodeling Integrates Morphological, Motility, and Cytokine Changes

Collagen remodeling was one of the most robust phenotypic alterations, particularly in the “Withdrawal” phase. This is evident when considering not only the visual changes in the collagen reorganization pattern, but also from the quantification, presented as collagen removal index (CRI). As there are 2 possible contributors to the CRI—total cell spreading area and total remodeling area it is important to discern their separate influence. According to the presented data (Table 1), it appears that the main contributor to changes in the value of CRI is the total remodeling area, rather than the cell spreading area. Notably, the differences in cell spreading are rather minimal, though maintaining the overall trend from Figure 2, likely due to the smaller statistical sample size, while the remodeling area differs significantly.

Both secretomes induced strong remodeling, but the temporal patterns differed—HG-3 triggered early remodeling, whereas RPMI-8226 induced delayed remodeling after withdrawal. This priming-dependent behavior aligns with the ECM-rich bone marrow niche of MM, where malignant plasma cells interact extensively with collagen I, collagen IV, and fibronectin [60]. CLL cells also interact with ECM through integrin-mediated adhesion [61], but their niche is more dynamic, which may explain the earlier but less amplified remodeling.

Importantly, collagen I is only one component of the ECM and does not fully represent the biochemical and mechanical diversity of the tumor microenvironment. ECM includes fibronectin, laminin, collagen IV, proteoglycans, all of which influence stromal behavior. Therefore, while collagen remodeling provides a robust functional readout, it should be interpreted as a partial representation of ECM-related changes.

The integration of spreading area, motility, and remodeling suggests a mechanobiological link: reduced spreading during exposure may reflect cytoskeletal contraction, whereas post-withdrawal expansion may increase traction forces, enabling stronger collagen compaction. Elevated bFGF and IL-6 levels further support this interpretation, as both cytokines promote cytoskeletal organization, motility, and ECM remodeling [18,56,61,62,63,64,65].

### 4.5. The AD-MSCs Response Differs from the Classical BM-MSCs Literature

Many of the observed phenotypes—senescence-associated activity, increased motility and enhanced remodeling—mirror tumor-induced changes previously described in BM-MSCs [29,32,66]. However, AD-MSCs possess distinct baseline properties, including higher proliferative capacity and different collagen interaction profiles. Our findings suggest that malignant B-cell secretomes can alter MSCs’ behavior across tissue sources. This raises the possibility that extramedullary adipose niches may contribute to disease progression in MM and CLL, consistent with reports of adipose-rich tissue involvement in both malignancies [67,68,69,70].

### 4.6. Significance of the Study

This study demonstrates that tumor-derived secretomes from two biologically distinct hematologic malignancies induce both shared and lineage-specific functional changes in AD-MSCs. By distinguishing immediate (“Exposure”) from persistent (“Withdrawal”) responses, we show that several tumor-driven phenotypes—particularly senescence-associated activity, G2/M accumulation, motility, and collagen remodeling—persist after secretome withdrawal, indicating early steps of stromal reprogramming. The elevated levels of IL-6 and bFGF detected in the conditioned media provide biochemical support for these coordinated responses. These findings highlight the capacity of malignant B-cell secretomes to reshape MSC behavior outside their native niche and underscore the importance of stromal plasticity in hematologic cancer progression.

### 4.7. Limitations of the Study and Future Perspectives

Several limitations should be considered when interpreting these findings.

First, the secretome was collected under serum-free conditions and concentrated using a 3 kDa cutoff, enriching for proteins while underrepresenting metabolites, lipids, and small peptides. The 72-h serum-free collection period represents a methodological compromise, and we cannot fully exclude an effect of serum deprivation on the composition of the collected secretome. Second, the Plain AD-MSC control includes concentrated RPMI medium, which does not fully reflect standard MSC culture conditions. Third, the “Exposure” and “Withdrawal” protocols begin with different initial MSC densities, which may influence proliferation and remodeling. Fourth, collagen I was used as a model ECM substrate. Future studies should incorporate fibronectin, laminin, collagen IV, proteoglycans, and matrix stiffness to capture the full complexity of the tumor ECM.

Future work should include proteomic and metabolomic characterization of MM and CLL secretomes, exploration of additional ECM components, and evaluation in 3D or co-culture systems that better mimic physiological stromal niches. Comparative studies across MSC tissue sources (BM-MSC, AD-MSC, UC-MSC) will further clarify lineage-specific stromal programming.

## 5. Conclusions

Collectively, our findings demonstrate that tumor-derived secretomes from two biologically distinct hematologic malignancies elicit both shared and lineage-specific functional alterations in AD-MSCs. By distinguishing immediate (“Exposure”) from persistent (“Withdrawal”) responses, we show that several tumor-driven phenotypes—including senescence-associated activity, G2/M accumulation, enhanced motility, and collagen remodeling—persist beyond the period of direct secretome contact, indicating early steps of stromal reprogramming. The elevated levels of IL-6 and bFGF detected in MSC-conditioned media further support the presence of an initial coordinated stromal activation program. Together, these observations highlight the ability of malignant B-cell secretomes to modulate MSC behavior outside their native niche and underscore the importance of stromal plasticity in the progression of hematologic cancers.

## Figures and Tables

**Table 1:** Quantitative comparison of collagen interaction metrics across “Exposure” and “Withdrawal” conditions. Measurements are acquired from the total fluorescent images of the sample. Data are presented as mean ± SD (*n* = 6). Statistical analysis was performed using one-way ANOVA followed by Tukey’s multiple comparisons test.

	Exposure			Withdrawal		
	Plain	RPMI	HG	Plain	RPMI	HG
Remodeled collagen area (×10^3^ µm^2^)	32.6 ± 6.3 ^ab^	25 ± 3.3 ^ac^	42.8 ± 4.3 ^bc^	24.8 ± 1.5 ^ab^	48.2 ± 4.9 ^a^	47 ± 2.6 ^b^
Cell spreading area (×10^3^ µm^2^)	284 ± 40.5	251.7 ± 17.3	244.9 ± 10.6	256.4 ± 17.7	273.7 ± 26.5	272.7 ± 22.9
CRI (Collagen Removal Index)	0.11 ± 0.02 ^a^	0.1 ± 0.02 ^b^	0.18 ± 0.02 ^ab^	0.1 ± 0.01 ^ab^	0.18 ± 0.02 ^a^	0.17 ± 0.02 ^b^

^a–c^ Different superscript letters within a row and condition (“Exposure”/“Withdrawal”) indicate statistically significant differences between groups (*p* ≤ 0.05).

## Data Availability

The original contributions presented in this study are included in the article. Further inquiries can be directed to the corresponding author.

## Supplementary Materials

The following supporting information can be downloaded at: https://www.mdpi.com/article/10.3390/cells15191775/s1, Figure S1: Quantile-quantile (Q-Q) plots of the mixed-model residuals for MSC cell-spreading area on a collagen substrate in response to RPMI-8226 and HG-3 secretome. (B) Residuals from the “Withdrawal” phase; Figure S2: FITC-collagen remodeling by AD-MSCs after exposure to cancer cell secretomes from myeloma (RPMI-8226) and leukemia (HG-3) cell lines. Representative fluorescence micrographs of AD-MSCs cultured for 5 h on FITC-collagen substrates under control conditions or after treatment with RPMI-8226 or HG-3-derived secretomes. Cells were analyzed under both “Exposure” (A) and “Withdrawal” (B) protocols. To visualize cell morphology, fixed samples were stained for actin (red) and nuclei (blue), overlaid on the fluorescent background of adsorbed FITC collagen (green). CellProfiler software was used to identify remodeled collagen regions (light blue) and superimpose them onto cell boundaries (orange outline).

## Abbreviations

The following abbreviations are used in this manuscript:

AD-MSCs	Human adipose-derived mesenchymal stem cells
bFGF	Fibroblast growth factor
BM-MSCs	Bone marrow–derived mesenchymal stem cells
CAFs	Cancer-associated fibroblasts
CA-MSCs	Cancer-associated mesenchymal stem cells
CCL	Chemokine (C–C motif) ligand
CI	Confidence interval
CLL	Chronic lymphocytic leukemia
CRI	Collagen removal index
CRI	Collagen removal index
DMSO	Dimethyl sulfoxide
ECM	Extracellular matrix
EMM	Estimated marginal means
FBS	Fetal bovine serum
FITC	Fluorescein isothiocyanate
IL	Interleukin
IQR	Interquartile range
miRNAs	Microribonucleic acid
MM	Multiple myeloma
MSCs	Mesenchymal stromal/stem cells
PBS	Phosphate-buffered saline
PFA	Paraformaldehyde
PI	Propidium iodide
RFU	Relative fluoresce units
ROIs	Regions of interest
RTC	Rat-tail tendon collagen
SASP	Senescence-associated secretory phenotype
SD	Standard deviation
SIPS	Stress-induced premature senescence
TAFs	Tumor-associated fibroblasts
TA-MSCs	Tumor-associated mesenchymal stem cells
TC	Tissue culture
TGF-β	Transforming growth factor beta
TME	Tumor microenvironment
VEGF	Vascular endothelial growth factor
